# Finerenone mitigates acute alcoholic myocardial injury by modulating inflammatory signaling, oxidative stress, and mitochondrial function

**DOI:** 10.1186/s10020-026-01532-2

**Published:** 2026-06-20

**Authors:** Ching-Han Liu, Tian-Huei Chu, Shih-Ming Yang, Yi-Chen Wang, Wen-Chin Lee, Yi-Chen Chang, Yao-Chang Chen, Yi-Jen Chen, Cheng-Chieh Fang, Zhi-Hong Wen, Chien-Wei Feng, Hao-Ming Wu, Po-Tseng Lee, Nan-Chieh Huang, Shih-Chung Huang

**Affiliations:** 1 Department of Internal Medicine, Division of Cardiology, Kaohsiung Armed Forces General Hospital, Kaohsiung, Taiwan; 2 Department of Internal Medicine, Division of Cardiology, Tri-Service General Hospital, National Defense Medical University, Taipei, Taiwan; 3 Department of Marine Biotechnology and Resources, National Sun Yat-Sen University, Kaohsiung, Taiwan; 4 School of Medicine, National Defense Medical University, Taipei, Taiwan; 5 Medical Laboratory, Medical Education and Research Center, Kaohsiung Armed Forces General Hospital, Kaohsiung, Taiwan; 6 Institute of Medical Science and Technology, National Sun Yat-Sen University, Kaohsiung, Taiwan; 7 Division of Plastic Surgery, Department of Surgery, Kaohsiung Medical University Hospital, Kaohsiung, Taiwan; 8 Department of Internal Medicine, Division of Nephrology, Kaohsiung Chang Gung Memorial Hospital and Chang Gung University College of Medicine, Kaohsiung, Taiwan; 9 Department of Biomedical Engineering, National Defense Medical University, Taipei, Taiwan; 10 Graduate Institute of Clinical Medicine, College of Medicine, Taipei Medical University, Taipei, Taiwan; 11 Division of Cardiology, Department of Internal Medicine, School of Medicine, College of Medicine, Taipei Medical University, Taipei, Taiwan; 12 Division of Cardiovascular Medicine, Department of Internal Medicine, Wan Fang Hospital, Taipei Medical University, Taipei, Taiwan; 13 Institute of BioPharmaceutical Sciences, National Sun Yat-Sen University, Kaohsiung, Taiwan; 14 National Museum of Marine Biology and Aquarium, Pingtung City, Pingtung County Taiwan; 15 Center for Cancer Research, Kaohsiung Medical University, Kaohsiung, Taiwan; 16 Office of Medical Education and Reasearch, Zuoying Armed Forces General Hospital, Kaohsiung, Taiwan; 17 Division of Cardiology, Department of Internal Medicine, College of Medicine, National Cheng Kung University Hospital, National Cheng Kung University, Tainan, Taiwan; 18 Diving Medical and Physiology Training Center, Zuoying Armed Forces General Hospital, Kaohsiung, Taiwan; 19 Department of Information Engineering, I-Shou University, Kaohsiung, Taiwan; 20 Department of Internal Medicine, Division of Cardiology, Pingtung Branch of Kaohsiung Armed Forces General Hospital, No. 22, Ln. 58, Dahu Rd., Pingtung City, Pingtung County 90092 Taiwan; 21 Department of Nursing, Fooyin University, Kaohsiung, Taiwan

**Keywords:** Acute alcoholic myocardial injury, Finerenone, Proinflammatory signaling, Oxidative stress, Mitochondria

## Abstract

**Background and purpose:**

Alcoholic cardiomyopathy (ACM) is a serious complication of chronic and acute alcohol abuse that can progress to heart failure (HF). Currently, no curative drug exists for ACM, and heart transplantation remains the only definitive option. Although mineralocorticoid receptor (MR) antagonists (MRAs) are a cornerstone of HF management, their role in ethanol cardiotoxicity remains poorly defined. Because ethanol elevates aldosterone levels, targeting the aldosterone/MR axis may represent a promising therapeutic strategy. This study investigated the effects of finerenone, a next-generation non-steroidal MRA, in both *in vitro* and *in vivo* models of acute alcoholic myocardial injury.

**Methods:**

H9c2 cardiomyocytes were exposed to ethanol with or without finerenone, and cell viability, apoptosis, mitochondrial dynamics, and proinflammatory signaling were assessed to evaluate cytoprotective effects. *In vivo*, a murine acute alcoholic myocardial injury model was generated by three days of ethanol exposure with concurrent finerenone administration. Circulating myocardial injury marker CK-MB was measured, and myocardial tissue was analyzed for MR/aldosterone levels, apoptosis, inflammation, macrophage infiltration, and mitochondrial fission/fusion dynamics.

**Results:**

Finerenone attenuated ethanol-induced MR upregulation, apoptosis, mitochondrial fragmentation, and pro-inflammatory signaling in H9c2 cardiomyocytes. In mice, finerenone further reduced the ethanol-induced increase in serum CK-MB and attenuated ethanol-induced myocardial cell death and damage. Moreover, ethanol-induced myocardial inflammation, oxidative stress, and mitochondrial dysfunction were further reduced in this mouse model of ethanol cardiotoxicity following finerenone administration. Together, the non-steroidal MRA finerenone may attenuate acute ethanol-induced cardiotoxicity by regulating inflammatory pathways, oxidative stress, and mitochondrial function.

**Conclusions and implications:**

Overall, these findings support the conclusion that the next-generation MRA finerenone attenuates acute ethanol-induced myocardial cell death, inflammation, oxidative stress, and mitochondrial dysfunction *in vitro* and *in vivo*. Finerenone may act as a novel protective agent against acute alcoholic myocardial injury in the future, but further clinical evidence is required to support this preclinical finding.

**Graphical Abstract:**

**Figure:**
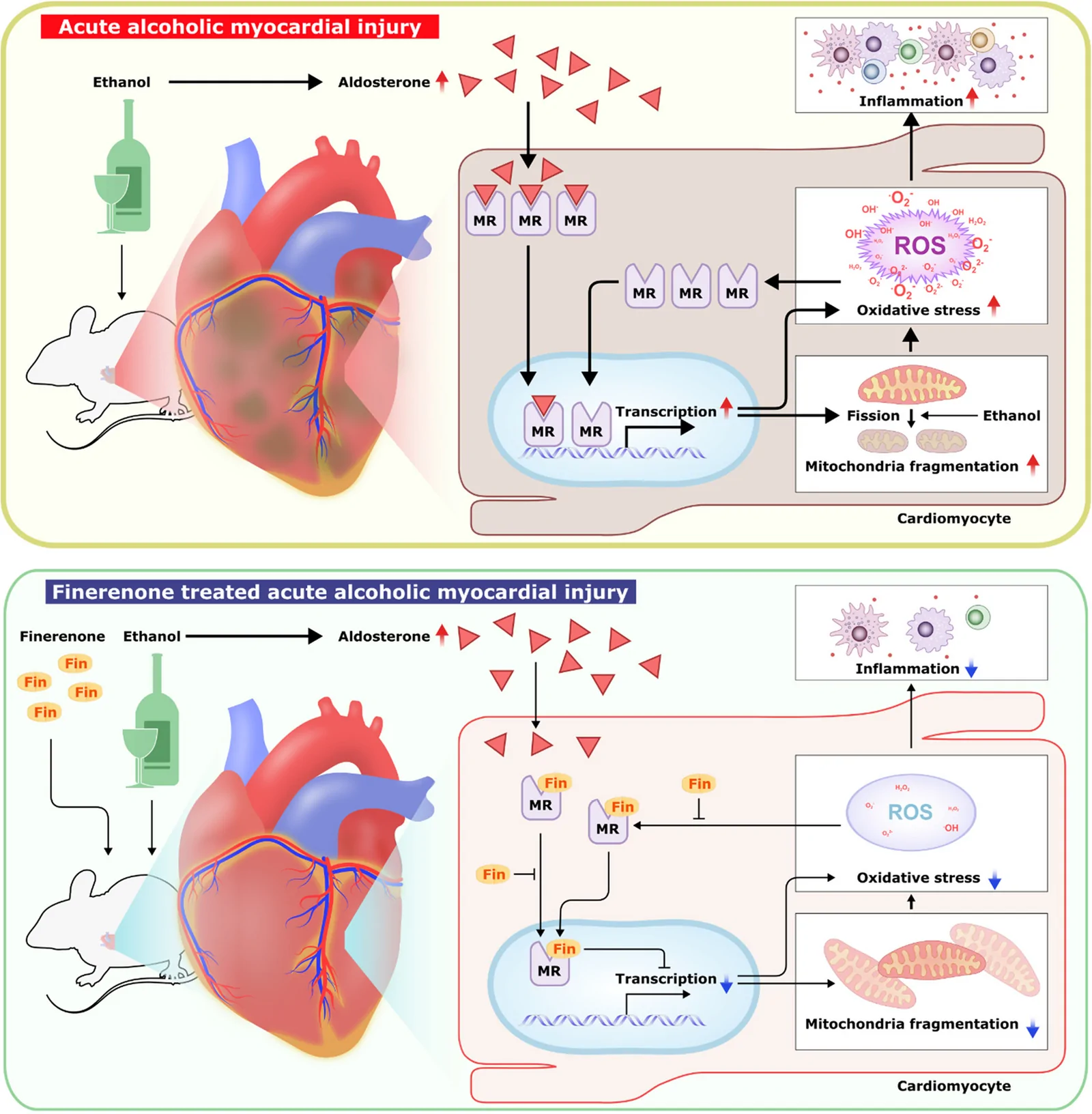

**Supplementary Information:**

The online version contains supplementary material available at https://doi.org/10.1186/s10020-026-01532-2.

## Highlights

- Finerenone attenuated ethanol-induced MR activation and cell death in cardiomyocytes.
- Finerenone blocked ethanol-promoted MR signaling and myocardial injury in mice.
- Ethanol-induced heart oxidative stress and inflammation were blocked by finerenone.
- Finerenone attenuated ethanol-induced mitochondrial dysfunction in mouse hearts.
- Finerenone may act as a potent cardioprotective agent against ethanol cardiotoxicity.

**Supplementary Information:**

The online version contains supplementary material available at https://doi.org/10.1186/s10020-026-01532-2.

## Introduction

Alcoholic cardiomyopathy (ACM) is a serious complication of chronic and acute alcohol abuse that can lead to heart failure (HF) (George and Figueredo 2011). Pathophysiologically, ACM can result from either acute binge drinking (acute ACM) or chronic alcohol consumption (chronic ACM) (Dominguez et al., 2024). Acute ACM is characterized by the acute toxic effects of ethanol on cardiomyocytes, often leading to atrial fibrillation and impaired cardiac contractility. This is typically presented as HF with reduced ejection fraction (HFrEF) (Sadhu et al., 2018). In contrast, chronic ACM involves the sustained production of reactive oxygen species (ROS), leading to irreversible mitochondrial dysfunction and chronic inflammation. These pathological processes ultimately lead to cardiac injury and fibrosis, culminating in dilated cardiomyopathy and the development of either HFrEF or HF with preserved ejection fraction (HFpEF) (Dominguez et al., 2024). Although acute and chronic ACM differ in their clinical presentations and specific pathophysiological pathways, they share common initial mechanisms, including ethanol-induced oxidative stress, mitochondrial dysfunction, and inflammation, which together drive cardiomyocyte damage and progressive cardiac dysfunction (Dominguez et al., 2024). At present, there is no curative pharmacological therapy for ACM, and heart transplantation remains the only definitive treatment. Therefore, further investigation into the underlying molecular mechanisms and the development of novel therapeutic strategies are critical for advancing ACM treatment.

The clinical use of mineralocorticoid receptor antagonists (MRAs) spans more than 60 years, primarily for the treatment of HF, hypertension, chronic kidney disease, primary aldosteronism, and post-myocardial infarction (Kolkhof et al., 2017). These therapeutic applications stem from MRAs' ability to block the effects of aldosterone within the renin–angiotensin–aldosterone system (RAAS), which regulates sodium retention, potassium excretion, and tissue remodeling (Kahlon 2022). Abnormal activation of RAAS has been implicated in the pathogenesis of HF (Kahlon et al., 2022), with aldosterone identified as a key contributor (Weber 2001; Chang et al., 2024). Accordingly, targeting aldosterone/MR signaling has shown therapeutic promise in HF management (Chang et al., 2024). While ethanol toxicity and its metabolites are widely recognized as key contributors to ACM pathology, the precise molecular mechanisms underlying this process remain incompletely understood. Some studies have reported that ethanol elevates serum aldosterone levels, which is associated with increased cardiovascular risk (Sobrino et al., 2019; Aoun et al., 2018). Given that MR activation promotes cardiac inflammation and injury (Young et al., 2015), ethanol-induced, aldosterone-dependent MR activation may play a pathogenic role in ACM. In addition, aldosterone-independent MR activation has also been implicated in cardiomyopathy development. For instance, Rac1, a non-classical upstream activator of MR, has been shown to mediate angiotensin II–induced cardiac fibrosis independently of aldosterone levels (Pan et al., 2024). Consistent with this, other studies have demonstrated that Rac1-mediated MR activation, independent of aldosterone, contributes to disease progression (Nagase and Fujita 2013). Spironolactone, as a steroidal MRA (25 to 50 mg/d), has been used to manage ACM patients with HF in New York Heart Association (NYHA) class II-IV (Nicolas et al., 2002). A clinical study published in 2016 showed the benefit of adding trimetazidine and carvedilol to traditional HF treatment, including spironolactone (Dominguez et al., 2024; Li et al., 2016). After a 12-week standard therapy including spironolactone, clinical data indicated that ACM patients receiving trimetazidine/carvedilol had a longer 6-min walking distance and higher left ventricular ejection fraction (Dominguez et al., 2024; Li et al., 2016). Collectively, these previous findings suggest that MR blockade, regardless of aldosterone levels, may have therapeutic value for ACM.

Finerenone, a next-generation non-steroidal MRA, exhibits greater selectivity for the MR and has a more favorable safety profile than earlier steroidal MRAs such as spironolactone and eplerenone (Kolkhof et al., 2017; Amazit et al., 2015). MR activation is known to stimulate NADPH oxidases (NOXs) and promote mitochondrial oxidative stress (Wu et al., 2018; Brandt et al., 2016). MRAs have been shown to attenuate aldosterone/MR signaling, thereby preserving mitochondrial integrity and function in cardiac tissue (Tsai et al., 2021; Hung et al., 2022). Recent studies have demonstrated that finerenone reduces the incidence of worsening HF events and all-cause mortality (Solomon et al., 2024; Chimura et al., 2024). However, the effects of finerenone in alcoholic myocardial injury have not been reported. Given the strong association between ACM and mitochondrial dysfunction (Piano and Phillips 2014), targeting MR using finerenone may be an effective strategy to mitigate ethanol-induced oxidative stress and mitochondrial damage.

In this study, we investigated the cardioprotective effects of finerenone against ethanol-induced cardiotoxicity using both H9c2 cardiomyocytes and a mouse model of acute myocardial injury. Our findings revealed that ethanol exposure significantly upregulated MR expression in both H9c2 cells and myocardial tissue, whereas finerenone treatment effectively suppressed this upregulation. Furthermore, finerenone alleviated ethanol-induced mitochondrial fission, oxidative stress, apoptosis, pro-inflammatory cytokine production, and macrophage infiltration. Collectively, these results suggest that finerenone may serve as a novel and effective cardioprotective agent against acute alcoholic myocardial injury.

## Materials and methods

### Cell culture

Rat H9c2 cardiomyocytes (ATCC, VA, USA) were maintained according to the manufacturer's instructions. All cell lines were incubated under humidified conditions, with 95% air and a 37˚C humidified incubator containing 5% CO_2_.

### Cell viability assay

H9c2 cells were cultured in 96-well plates. After treatment, cells were washed with PBS and incubated in conditioned medium containing alamarBlue™ (Thermo Fisher Scientific, MA, USA) at 37 °C for 2 h to allow resazurin reduction to resorufin. The conditioned medium was then transferred to a new 96-well plate, and resorufin absorbance was measured at 570 nm with a reference wavelength of 620 nm using a microplate reader.

### Animals

Adult male FVB mice were obtained from BioLASCO Co., Ltd. (Taipei, Taiwan). All mice were maintained and bred in specific pathogen-free animal facilities, where they received water and commercial rat food ad libitum under a 12/12 h light/dark cycle. The protocol of the animal study was reviewed and approved by the Institutional Animal Care and Use Committee at Kaohsiung Armed Forces General Hospital (No. A114-7). All animals received humane care based on the Animal Research: Reporting *In Vivo* Experiments (ARRIVE) guidelines and the Guide for the Care and Use of Laboratory Animals published by the National Institutes of Health (NIH Publication, 8th edition, 2011). During the experiment, we strictly followed the guidelines for pain experiments in awake animals.

### Acute alcoholic myocardial injury model and treatments

In this study, an acute ethanol-induced myocardial injury mouse model (3 g/kg/d for 3 days via intraperitoneal injection) was used to investigate the cardioprotective effect of finerenone (Ma et al., 2009; Guo et al., 2010a; Guo and Ren 2012a; Tao et al., 2021; Deng et al., 2021). Based on previous preclinical studies, oral finerenone at 10 mg/kg/d has a cardioprotective effect in various mouse cardiovascular disease models (Grune et al., 2016, 2018; Morikawa et al., 2026). Mice were randomly divided into four groups (*n* = 5 per group): [1] Control group (equal-volume normal saline by intraperitoneal injection + equal-volume drinking water by oral gavage); [2] Ethanol group (ethanol by intraperitoneal injection + equal-volume drinking water by oral gavage); [3] Finerenone group (equal-volume normal saline by intraperitoneal injection + finerenone by oral gavage); and [4] Ethanol + finerenone group (ethanol by intraperitoneal injection + finerenone by oral gavage). Therefore, adult male FVB mice, 6 weeks old, received ethanol (3 g/kg/d) through three discontinuous intraperitoneal (i.p.) injections with isotonic 20% ethanol per day for a 3-day treatment on day 0, along with finerenone (10 mg/kg) delivered through oral gavage once a day for a 3-day treatment. The mice received either an i.p. injection of normal saline or an oral gavage of drinking water, which served as treatment control. Mice are sacrificed on day 3, and the specimens, including sera and heart tissues, are collected for further analysis. Before animal sacrificing, blood samples for biochemical analysis were collected from the submandibular vein using a sterile lancet. Then the animals were euthanized by exsanguination under deep and sustained anesthesia (5% isoflurane, 0.5 L/min) in this study. A portion of the collected heart tissue was stored in a −80 °C freezer for further immunoblotting analysis, while the remaining tissue samples were fixed in 4% paraformaldehyde for subsequent processing and embedding. To ensure unbiased evaluation, tissue harvesting, histological staining quantification, and biochemical measurements were performed by investigators blinded to the treatment groups.

### CK-MB detection analysis

Serum samples obtained from the mouse ACM model were analyzed for CK-MB levels using Beckman Coulter CK-MB reagent kit (OSR61155; Beckman Coulter, Inc., CA, USA), performed on Beckman Coulter DxI 800 Immunoassay Analyzer (Beckman Coulter, Inc., CA, USA) in the Department of Laboratory Medicine, Kaohsiung Armed Forces General Hospital.

### TUNEL assay

For H9c2 cells, coverslips were placed in 6-well plates, and cells were seeded accordingly. After treatment, cells were fixed with 4% paraformaldehyde and permeabilized in TBST containing 1% BSA and 0.2% Triton X-100. For the myocardial tissue obtained from the ACM model, paraffin-embedded samples were sectioned at 3 μm thickness and permeabilized in TBST with 1% BSA and 0.2% Triton X-100. TUNEL staining was performed according to the manufacturer’s protocol (S7110; Merck Millipore, Billerica, MA, USA). Nuclear counterstaining was performed with DAPI. Fluorescent signals of DNA fragmentation were visualized with a fluorescence microscope (IX51; Olympus, Tokyo, Japan).

### Immunofluorescence and immunohistochemical analyses

Paraffin-embedded left ventricle tissues were sliced to 3 μm and permeabilized in TBST containing 1% BSA and 0.2% Triton X-100. For immunofluorescence staining, the sections were incubated overnight with primary antibodies, followed by incubation with fluorophore-conjugated secondary antibodies and nuclear counterstaining with DAPI. For immunohistochemical staining, the sections were permeabilized in TBST containing 1% BSA and 0.2% Triton X-100, then incubated with primary antibody and HRP-labeled secondary antibody. The signal was developed using DAB, and hematoxylin staining was performed to visualize the nucleus. The heart section was covered with mounting media and visualized under a microscope. In this study, four mouse hearts per group were performed immunohistochemical analysis, and each heart section was photographed in three views using a microscope equipped with a digital imaging system (Olympus Corporation, Tokyo, Japan) for further quantification (Yin et al., 2025; Goldsmith et al., 2020). The density and intensity of the signal were determined by ImageJ software (NIH, USA). Commercial antibodies and their dilution factors used for histological analysis are as follows: Aldosterone (ARG23497, 1:500) (Arigo Biolaboratories, Hsinchu, Taiwan); MR (21,854–1-AP, 1:200) (Proteintech, Rosemont, IL); iNOS (18,985–1-AP, 1:500) (Proteintech, Rosemont, IL); F4/80 (#70,076; 1:250) (Cell Signaling, Beverly, MA); 8-hydroxy-2-deoxyguanosine (8-OHdG) (sc-66036; 1:100) (Santa Cruz Biotechnology, Santa Cruz, CA); MFN1 (ab104585, 1:300) (Abcam, Cambridge, UK); FIS1(sc-98900, 1:200) (Santa Cruz Biotechnology, Santa Cruz, CA).

### Immunoblotting analysis

Tissue and cells were homogenized in RIPA buffer containing protease and phosphatase inhibitors (Roche, Basel, Switzerland). The protein extract was separated by electrophoresis on an SDS-PAGE gel (Bio-Rad, Burlington, MA, USA) and transferred onto a PVDF transfer membrane. Following blocking with 5% skim milk, the membrane was incubated with primary antibody and HRP-conjugated secondary antibody. Signals were visualized and exposure on the KETA gel documentation system (Neo-One Bio Life Science, Taipei, Taiwan). The density and intensity of the signal will be determined by the ImageJ software (NIH, USA). Commercial antibodies and their dilution factors used for immunoblotting are as follows: Cleaved caspase-3 (#9664, 1:1000) (Cell Signaling, Beverly, MA); MR (21,854–1-AP, 1:1000) (Proteintech, Rosemont, IL); iNOS (A3774, 1:1000) (ABclonal Technology, Woburn, MA); IL-1β (16,806–1-AP, 1:1000) (Proteintech, Rosemont, IL); TNF-α (GTX110520, 1:1000) (GeneTex, Irvine, CA); HMGB1 (ab18256,1:1000) (Abcam, Cambridge, UK); p-NFκB p65 (Ser536) (sc-136548, 1:500) (Santa Cruz Biotechnology, Santa Cruz, CA); NFκB p65 (sc-8008, 1:500) (Santa Cruz Biotechnology, Santa Cruz, CA); FIS1 (sc-98900, 1:500) (Santa Cruz Biotechnology, Santa Cruz, CA); MFN1 (ab104585, 1:1000) (Abcam, Cambridge, UK); MFN2 (sc-100560, 1:500) (Santa Cruz Biotechnology, Santa Cruz, CA); β-actin (sc-47778, 1:500) (Santa Cruz Biotechnology, Santa Cruz, CA); GAPDH (ARG10112, 1:3000) (Arigo Biolaboratories, Hsinchu, Taiwan).

### Mitochondria fragmentation analysis

H9c2 cells were seeded on coverslips placed in 6-well plates. After treatment, cells were incubated with fresh conditioned medium containing 100 nM MitoTracker™ Red CMXRos (M7512; Invitrogen, Carlsbad, CA) at 37 ℃ for 30 min. The cells were then rinsed three times with conditioned medium and fixed with 4% paraformaldehyde. Fluorescence signals were visualized using a fluorescence microscope (IX51; Olympus, Tokyo, Japan), and mitochondrial fragmentation ratios were quantified using ImageJ software (NIH, USA).

### Statistics

The sample size for the present study was calculated using G*Power analysis based on predicted large effect size (*f* = 0.45), an alpha level of 0.05, and a power of 0.80 (Faul et al., 2007). All experiments were performed in triplicate at a minimum. Before statistical analysis, data were assessed for normality using the Shapiro–Wilk test. When necessary, logarithmic transformation (log_10_(Y)) was applied to improve data distribution (Motulsky et al., 2025). Statistical comparisons were then performed using one-way ANOVA or two-way ANOVA followed by Tukey's post hoc tests. If zero values were present in the dataset, the data were transformed using a logarithmic function, log_10_(Y + 1), to achieve normality and homogeneity of variance before further analysis (Osborne 2002). The statistical results are shown as mean ± SD, and *P* < 0.05 was considered statistically significant. GraphPad Prism 10 (GraphPad Software, San Diego, CA) was used for the statistical calculations in the present study. Quantitative analysis of immunoblotting and histopathological data was performed using ImageJ software (NIH), with relative gene expression levels in the control group defined as 1.

## Results

### Finerenone mitigated ethanol-induced cytotoxicity in H9c2 cardiomyocytes

**Fig. 1:**
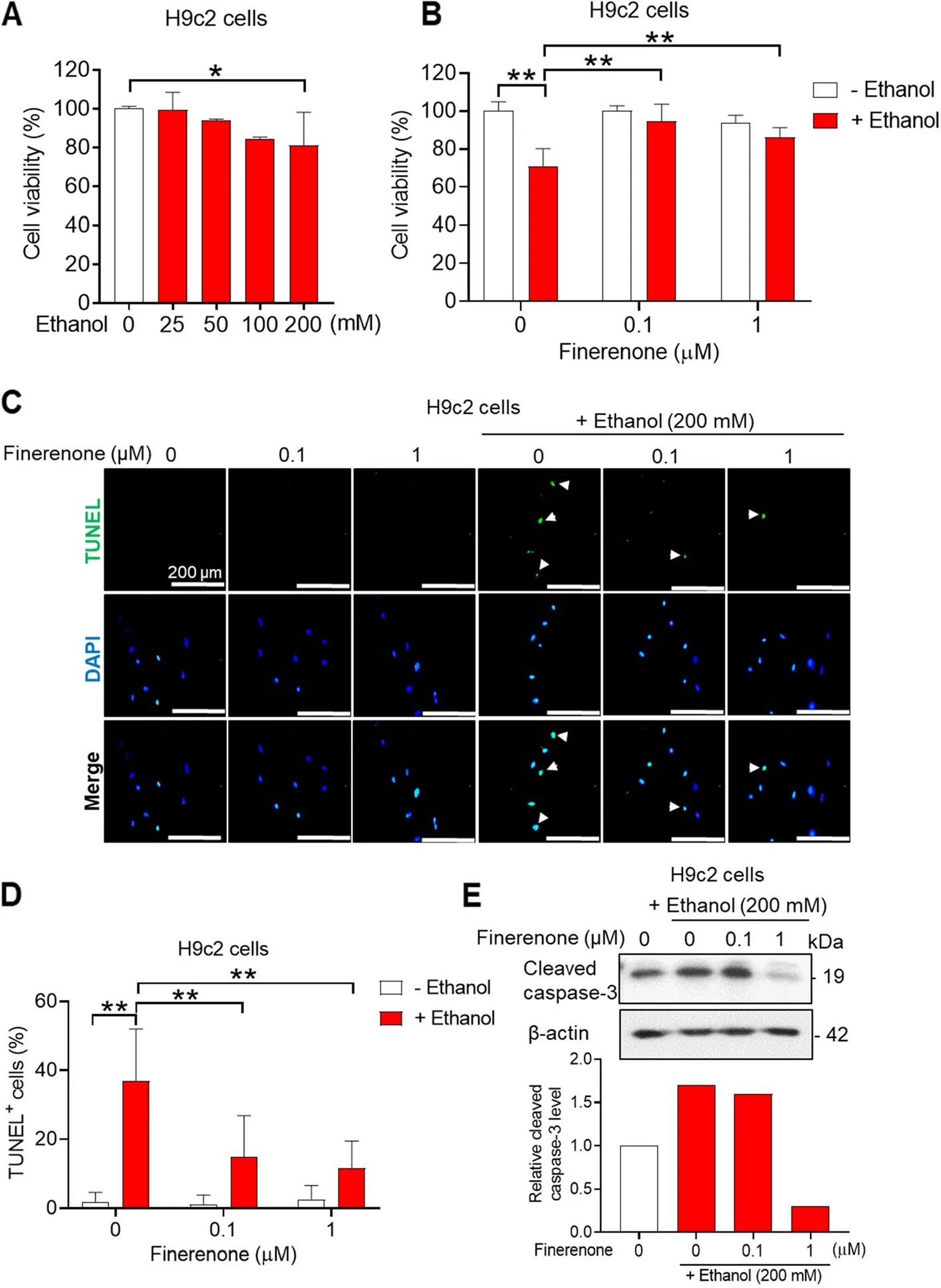
Finerenone attenuated ethanol-induced cytotoxicity in H9c2 cardiomyocytes. **A** H9c2 cells were treated with various concentrations of ethanol (0–200 mM) for 24 h, and cell viability was assessed using the Alamar Blue assay (*n* = 4 in each group). Mean ± SD. *P < 0.05; one-way ANOVA. **B** H9c2 cells were treated with finerenone (0.1 and 1 μM) alone or in combination with ethanol (200 mM) for 24 h, and cell viability was assessed using the Alamar Blue assay (*n* = 7 in each group). Mean ± SD. *P < 0.05, **P < 0.01; two-way ANOVA. **C** H9c2 cells treated with finerenone (0.1 and 1 μM) alone or in combination with ethanol (200 mM) for 24 h were subjected to TUNEL staining. Scale bar = 200 μm. **D** Bar graph shows the number of TUNEL^+^ cells per image field (*n* = 6 in each group). Mean ± SD. **P < 0.01; two-way ANOVA. (E) H9c2 cells treated with finerenone (0.1 and 1 μM) alone or in combination with ethanol (200 mM) for 24 h were subjected to immunoblotting analysis for cleaved-caspase-3 levels. Bar graph shows the relative cleaved-caspase-3 level

To evaluate the cardioprotective effects of finerenone against ethanol exposure, we first assessed the viability of H9c2 cells after dose-escalation with ethanol (0–200 mM). Ethanol (50–200 mM) significantly reduced the viability of H9c2 cells (Fig. 1A). Cleaved caspase-3, as an apoptosis marker, was upregulated in the ethanol-treated H9c2 cells (Supplementary Fig. 1). Treatment with finerenone (0.1 and 1 μM) alone had no impact on cell viability. However, finerenone (0.1 and 1 μM) significantly attenuated ethanol (200 mM) induced reduction in cell viability in H9c2 cells (Fig. 1B). Further evaluating the ability of finerenone to mitigate ethanol-induced decreases in cell viability, apoptosis was assessed using TUNEL staining and immunoblotting for the expression of cleaved caspase-3. The results showed that finerenone treatment alone did not increase the number of TUNEL^+^ cells (Fig. 1C and D). Treatment with 200 mM ethanol significantly increased the number of TUNEL^+^ cells; however, this increase was significantly attenuated by finerenone (0.1 and 1 μM) treatments (Fig. 1C and D). Further analysis of cleaved caspase-3 expression following combined treatment with ethanol and finerenone revealed that the ethanol-induced elevation of cleaved caspase-3 expression was attenuated by finerenone (0.1 and 1 μM) (Fig. 1E). These findings suggest that MR blockade with finerenone provides cardioprotection against ethanol-induced cytotoxicity.

### Finerenone protected cardiomyocytes from acute ethanol-induced cardiac damage in mice

**Fig. 2:**
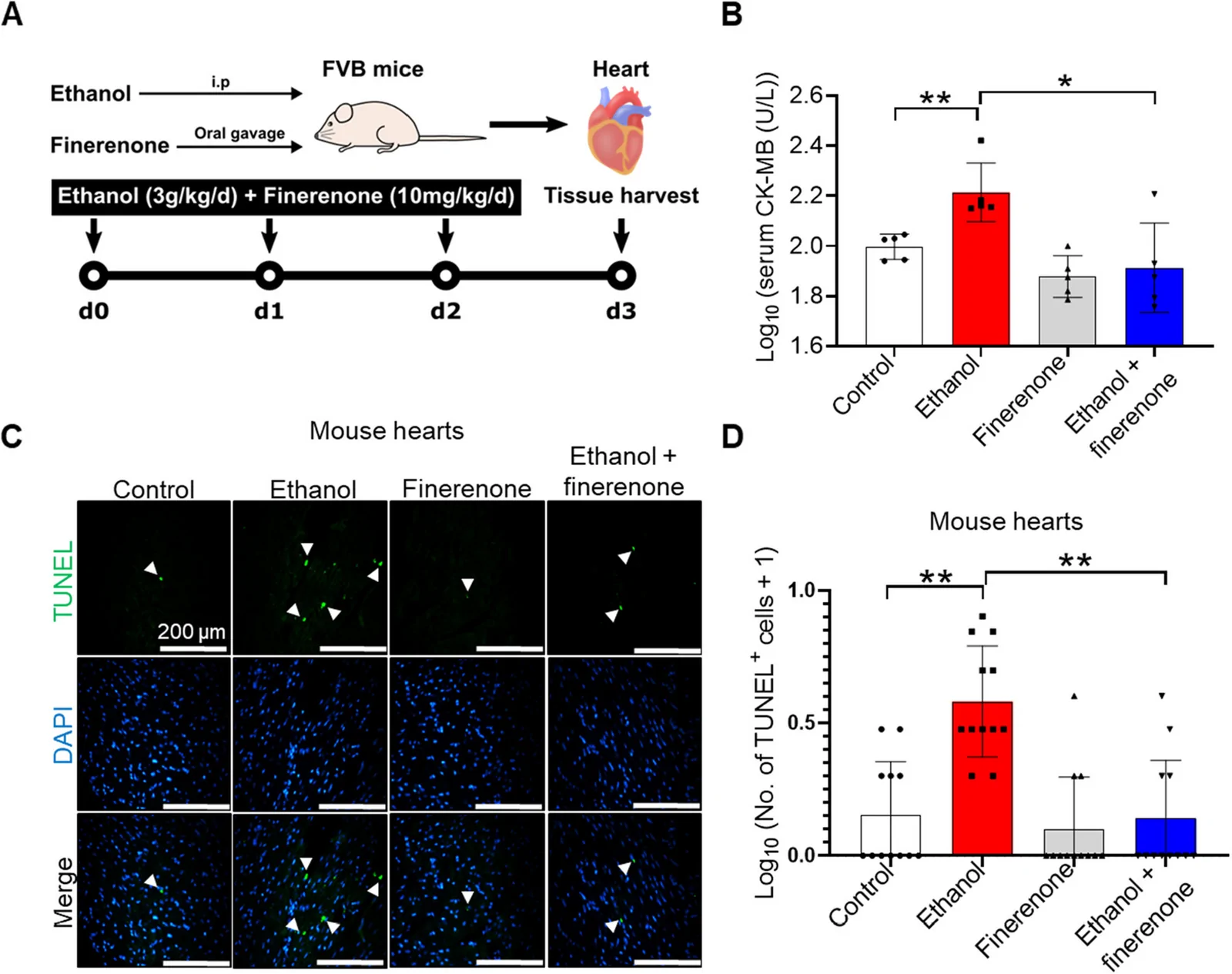
Finerenone attenuated ethanol-induced myocardial damage in a mouse acute alcoholic myocardial injury model. **A** Schematic representation of the acute ethanol exposure model in FVB mice. Mice received intraperitoneal injections of ethanol (3 g/kg) once daily for three consecutive days. For finerenone treatment, mice were administered finerenone (10 mg/kg) via oral gavage once daily for three consecutive days. Mice were sacrificed, and tissues were collected on day 3. **B** Serum CK-MB concentration was measured to assess myocardial injury in all mice (*n* = 5 per group), and Log_10_ (serum CK-MB (U/L)) was shown in the bar graph. Mean ± SD. *P < 0.05, **P < 0.01; two-way ANOVA. **C** Myocardial tissue was subjected to TUNEL staining to evaluate apoptosis. Scale bar = 200 μm. **D** Log_10_ (No. of TUNEL^+^ cells + 1) per image field was shown in the bar graph. n = 12 (four mice in each group, three images were analyzed for each mouse heart). Mean ± SD. **P < 0.01; two-way ANOVA

The *in vitro* results described above demonstrate that finerenone prevents ethanol-induced inflammation, apoptosis, and mitochondrial dysfunction in H9c2 cardiomyocytes. To further evaluate the cardioprotective effects of finerenone *in vivo*, we utilized a mouse model of acute alcoholic myocardial injury as previously described, and FVB mice are susceptible to alcohol exposure (Shah et al., 2010). In this model, FVB mice were administered ethanol (3 g/kg, intraperitoneally) and finerenone (10 mg/kg, oral gavage) once daily for three consecutive days. Mice were sacrificed on day four post-ethanol challenge and finerenone treatment (Fig. 2A). Results revealed that acute ethanol exposure significantly elevated the cardiac damage marker, serum CK-MB levels (Fig. 2B). Treatment with finerenone markedly attenuated the ethanol-induced increase in serum CK-MB levels (Fig. 2B). Furthermore, TUNEL staining of myocardial tissue showed a significant increase in TUNEL-positive cells following ethanol exposure, which was significantly attenuated by finerenone treatment (Fig. 2C and D).

### Finerenone attenuated ethanol-promoted MR signaling and proinflammatory pathway in H9c2 cardiomyocytes

**Fig. 3:**
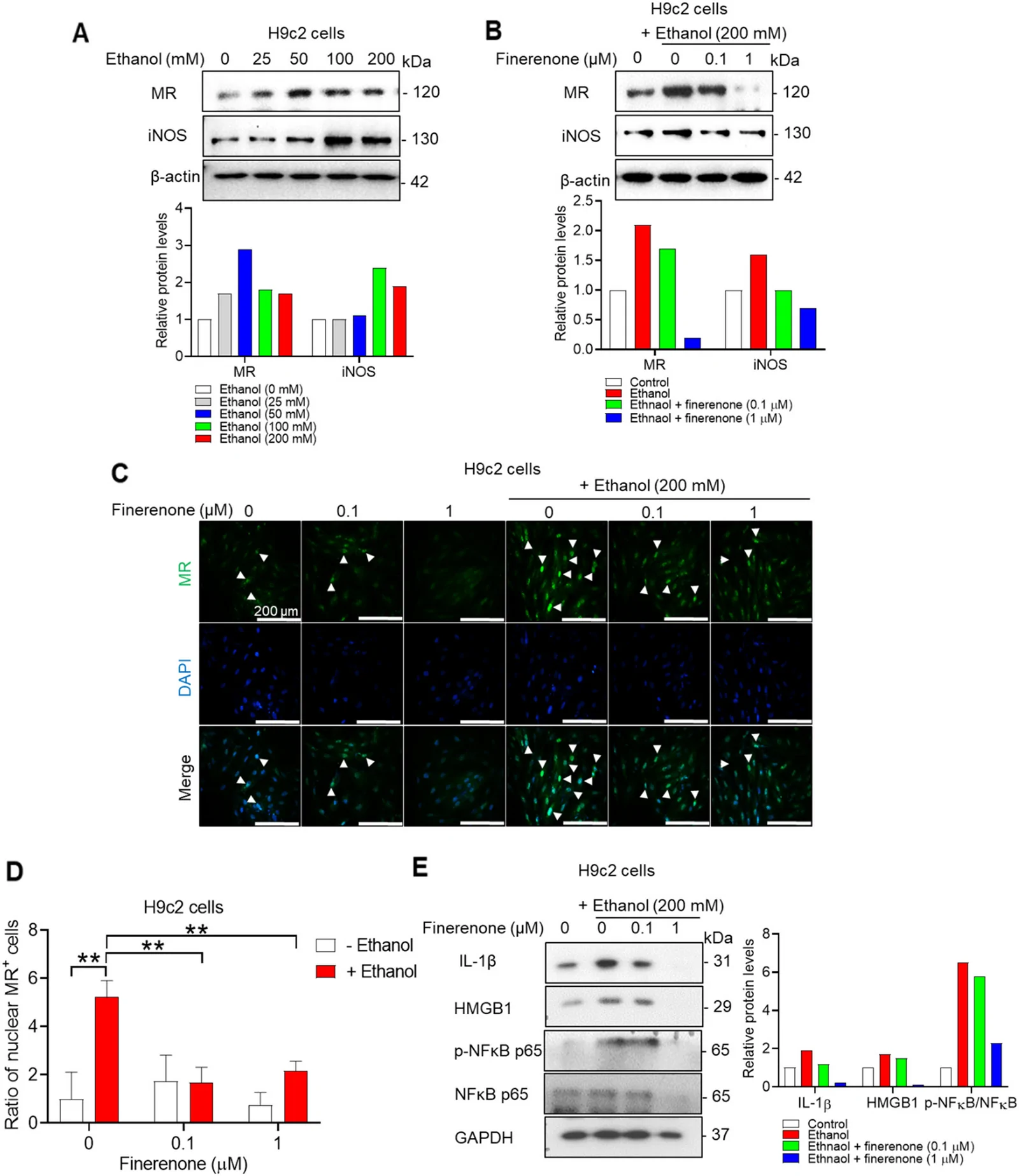
Finerenone blocked ethanol-promoted MR signaling and proinflammatory pathway in H9c2 cardiomyocytes. **A** Protein lysates from H9c2 cells treated with various concentrations of ethanol (0–200 mM) were analyzed by immunoblotting to assess MR and iNOS expressions. Bar graph shows the relative MR and iNOS levels. The experiments of Figs. 3A and 7C were performed using the same sample and blot, and the identical β-actin bands are shown in these two panels. **B** H9c2 cells treated with finerenone (0.1 and 1 μM) alone or in combination with ethanol (200 mM) for 24 h were subjected to immunoblotting analysis for MR and iNOS expression. Bar graph shows the relative MR and iNOS levels. **C** H9c2 cells treated with finerenone (0.1 and 1 μM) alone or in combination with ethanol (200 mM) for 24 h were subjected to immunofluorescence staining to evaluate the number of nuclear MR^+^ cells. Scale bar = 200 μm. **D** Bar graph shows the ratio of nuclear MR^+^ cells per image field. DAPI counterstain was used to visualize the nucleus (*n* = 3 per group). Mean ± SD. **P < 0.01; two-way ANOVA. **E** H9c2 cells treated with finerenone (0.1 and 1 μM) alone or in combination with ethanol (200 mM) for 24 h were subjected to immunoblotting analysis for IL-1β, HMGB1, p-NFκB p65, and NFκB p65 levels. Bar graph shows relative IL-1β/β-actin, HMGB1/β-actin, and p-NFκB p65/p65 ratios

Our present results showed that finerenone mitigated ethanol-induced cardiomyocyte damage, suggesting that ethanol modulates the MR involved in ethanol cardiotoxicity. To further investigate the role of MR implicated in ethanol-induced cardiac damage, we examined MR expression following ethanol challenge. Ethanol exposure (100 and 200 mM) increased MR expression in H9c2 cardiomyocytes (Fig. 3A). Further validation confirmed that ethanol exposure (100 and 200 mM) promoted cardiac inflammation as well as increased iNOS expression in H9c2 cardiomyocytes (Fig. 3A). Thus, we employed finerenone treatment in conjunction with ethanol exposure. Results showed that finerenone suppressed ethanol-induced upregulation of MR and iNOS in H9c2 cardiomyocytes (Fig. 3B). MR activation involves the nuclear translocation of MR, enabling it to function as a transcription factor (Bauersachs et al., 2015). Further analysis using immunofluorescence staining revealed that exposure to ethanol (200 mM) significantly increased the number of nuclear MR^+^ H9c2 cardiomyocytes (Fig. 3C and D). MR blockade with finerenone (0.1 and 1 μM) significantly mitigated the ethanol-induced increase in the number of nuclear MR^+^ H9c2 cardiomyocytes (Fig. 3C and D). Further examination of ethanol-induced inflammatory factors revealed that ethanol administration (25–200 mM) promoted IL-1β and HMGB1 overexpression in H9c2 cells (Supplementary Fig. 2). Furthermore, finerenone attenuated the ethanol-induced upregulation of IL-1β and HMGB1 and mitigated the ethanol-induced increase in the p-NFκB p65/p65 ratio in H9c2 cardiomyocytes (Fig. 3E).

### Finerenone attenuated the acute ethanol-promoted MR activation and cardiac inflammation in mouse hearts

**Fig. 4:**
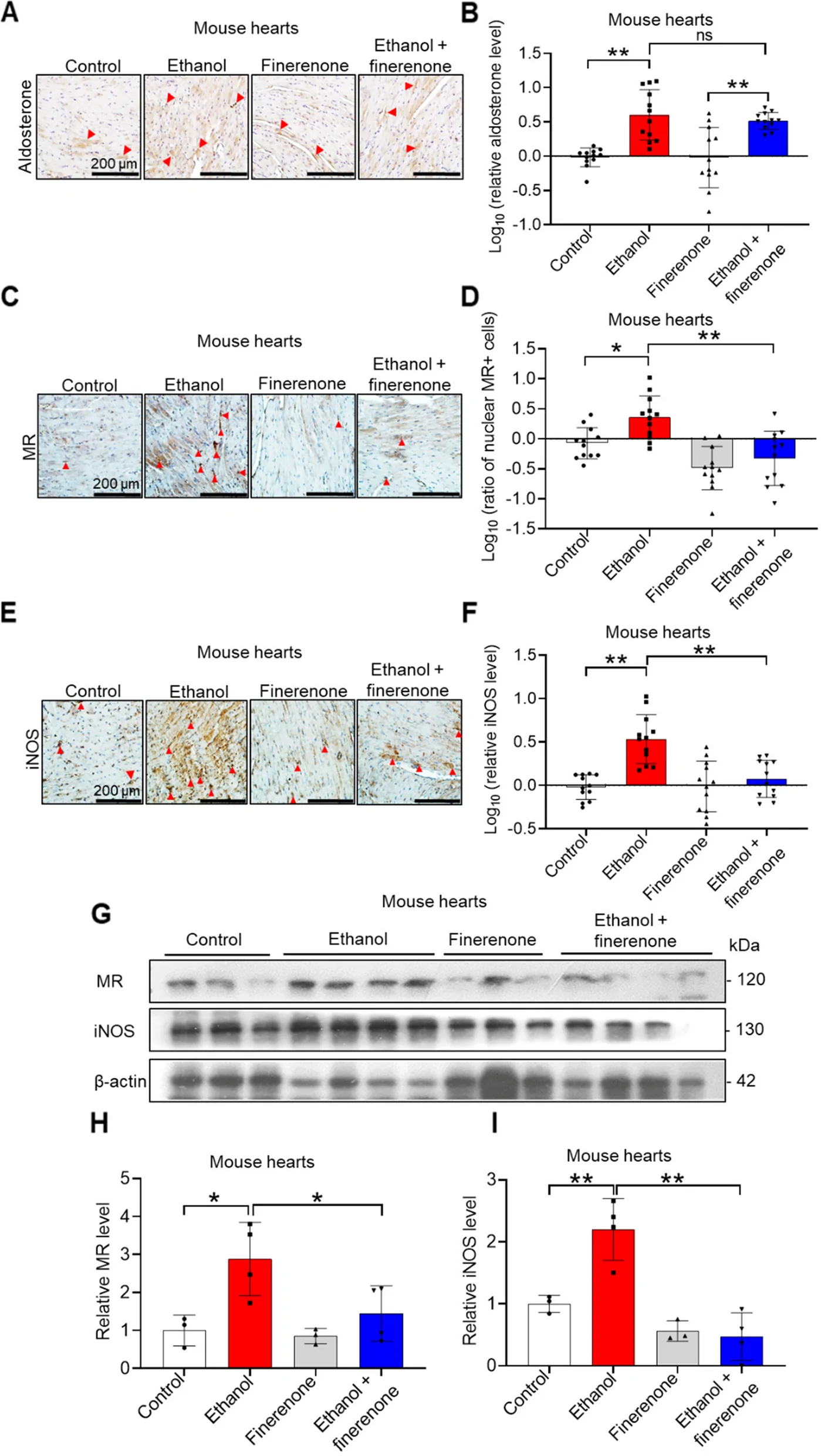
Finerenone attenuated the acute ethanol-promoted MR and iNOS pathways in mouse hearts. Myocardial tissue from mice treated with ethanol ± finerenone was analyzed by immunohistochemical staining to detect cardiac expression of (**A** and **B**) aldosterone, (**C** and **D**) MR, and (**E** and **F**) iNOS. Scale bar = 200 μm. Bar graphs show (**B**) Log_10_ (relative aldosterone level), (**D**) Log_10_ (ratio of nuclear MR^+^ cells), and (**F**) Log_10_ (relative iNOS level), respectively. n = 12 (four mice in each group, three images were analyzed for each mouse heart). Mean ± SD. *P < 0.05, **P < 0.01, ns = not significant; two-way ANOVA. **G** The immunoblotting analysis of MR and iNOS expression was performed in mouse hearts after ethanol administration ± finerenone. 3–4 mouse hearts per group were used for immunoblotting. **H** The bar graph shows relative MR levels (*n* = 3 in the control group, *n* = 4 in the ethanol group, *n* = 3 in the finerenone group, and *n* = 4 in the ethanol + finerenone group). Mean ± SD. *P < 0.05; two-way ANOVA. **I** The bar graph shows relative iNOS levels (*n* = 3 in the control group, *n* = 4 in the ethanol group, *n* = 3 in the finerenone group, and *n* = 4 in the ethanol + finerenone group). Mean ± SD. **P < 0.01; two-way ANOVA

**Fig. 5:**
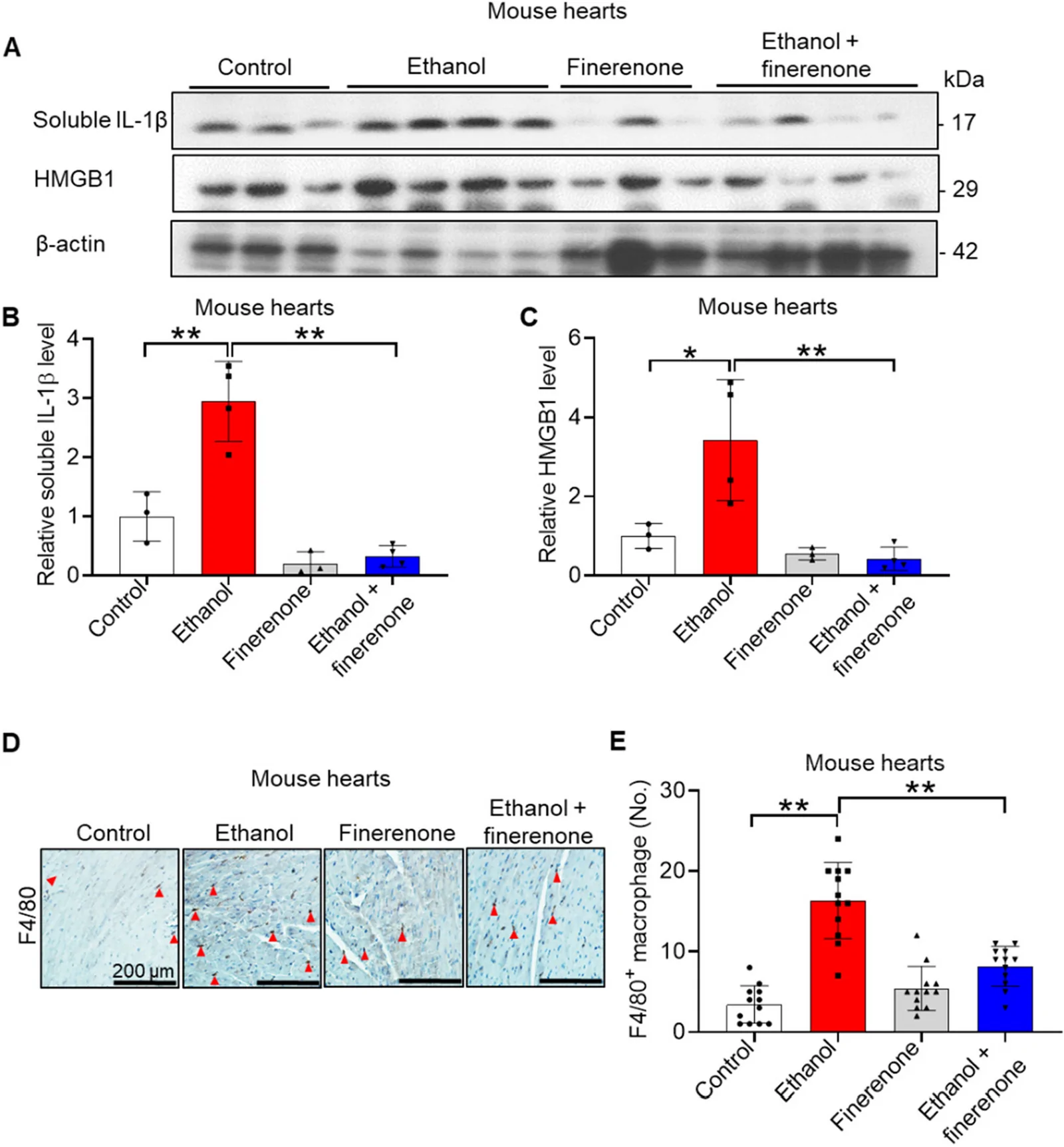
Oral finerenone blocked the acute alcohol-promoted myocardial inflammation in mice. **A** The immunoblotting analysis of soluble IL-1β and HMGB1 expression was performed in mouse hearts after ethanol administration ± finerenone. 3–4 mouse hearts per group were used for immunoblotting. **B** Bar graph shows the relative soluble IL-1β levels (*n* = 3 in the control group, *n* = 4 in the ethanol group, *n* = 3 in the finerenone group, and *n* = 4 in the ethanol + finerenone group). Mean ± SD. **P < 0.01; two-way ANOVA. **C** Bar graph shows relative HMGB1 levels (*n* = 3 in the control group, *n* = 4 in the ethanol group, *n* = 3 in the finerenone group, and *n* = 4 in the ethanol + finerenone group). Mean ± SD. *P < 0.05, **P < 0.01; two-way ANOVA. **D** Representative images show immunohistochemical staining for F4/80 in mouse hearts following ethanol treatment, with or without finerenone. Scale bar = 200 μm. **E** Bar graph shows the number of F4/80^+^ cells per image field. *n* = 12 (four mice in each group, three images were analyzed for each mouse heart). Mean ± SD. **P < 0.01; two-way ANOVA

Our *in vitro* results showed that finerenone attenuated ethanol-induced MR upregulation and inflammation. To evaluate the cardioprotective effects of finerenone on ethanol-induced aldosterone and MR levels, heart tissue obtained from the mouse model of acute alcoholic myocardial injury was analyzed by immunohistochemistry for aldosterone, MR, and iNOS expression. Results showed that ethanol significantly increased aldosterone levels in the myocardial tissue (Fig. 4A and B). Finerenone treatment mildly decreased ethanol-induced cardiac aldosterone levels; however, no significant difference was observed (Fig. 4A and B). Further examination of MR expression in the mouse hearts revealed that finerenone treatment significantly attenuated ethanol-induced nuclear MR localization (Fig. 4C and D). Finerenone also attenuated ethanol-induced upregulation of the proinflammatory iNOS in mouse myocardial tissues (Fig. 4E and F). Moreover, MR and iNOS protein levels were further validated by immunoblotting, and MR and iNOS were significantly upregulated in ethanol-treated mouse hearts compared with the control group (Fig. 4G-I). Oral finerenone significantly blocked the alcohol-stimulated MR and iNOS pathways in mouse myocardial tissues (Fig. 4G-I). In cell experiments, finerenone potently attenuated ethanol-induced overproduction of IL-1β and HMGB1 in H9c2 cardiomyocytes. Thus, the effect of finerenone on IL-1β/HMGB1 signaling was further investigated in ethanol-treated mouse cardiac tissues. The immunoblotting analysis showed that soluble IL-1β and HMGB1 were significantly upregulated in ethanol-treated mouse hearts compared with the control group (Fig. 5A-C), and that oral finerenone significantly attenuated ethanol-induced IL-1β/HMGB1 signaling (Fig. 5A-C). Besides, the immunoblotting analysis also showed that TNF-α signaling in ethanol-treated mouse hearts was significantly blocked by finerenone (Supplementary Fig. 3). Further examination of inflammation-promoted macrophage infiltration revealed that ethanol significantly increased the accumulation of F4/80^+^ cells in the mouse myocardial tissues, whereas finerenone significantly attenuated this increase (Fig. 5D and E).

### Finerenone mitigated ethanol-induced oxidative damage in the cardiac tissue

**Fig. 6:**
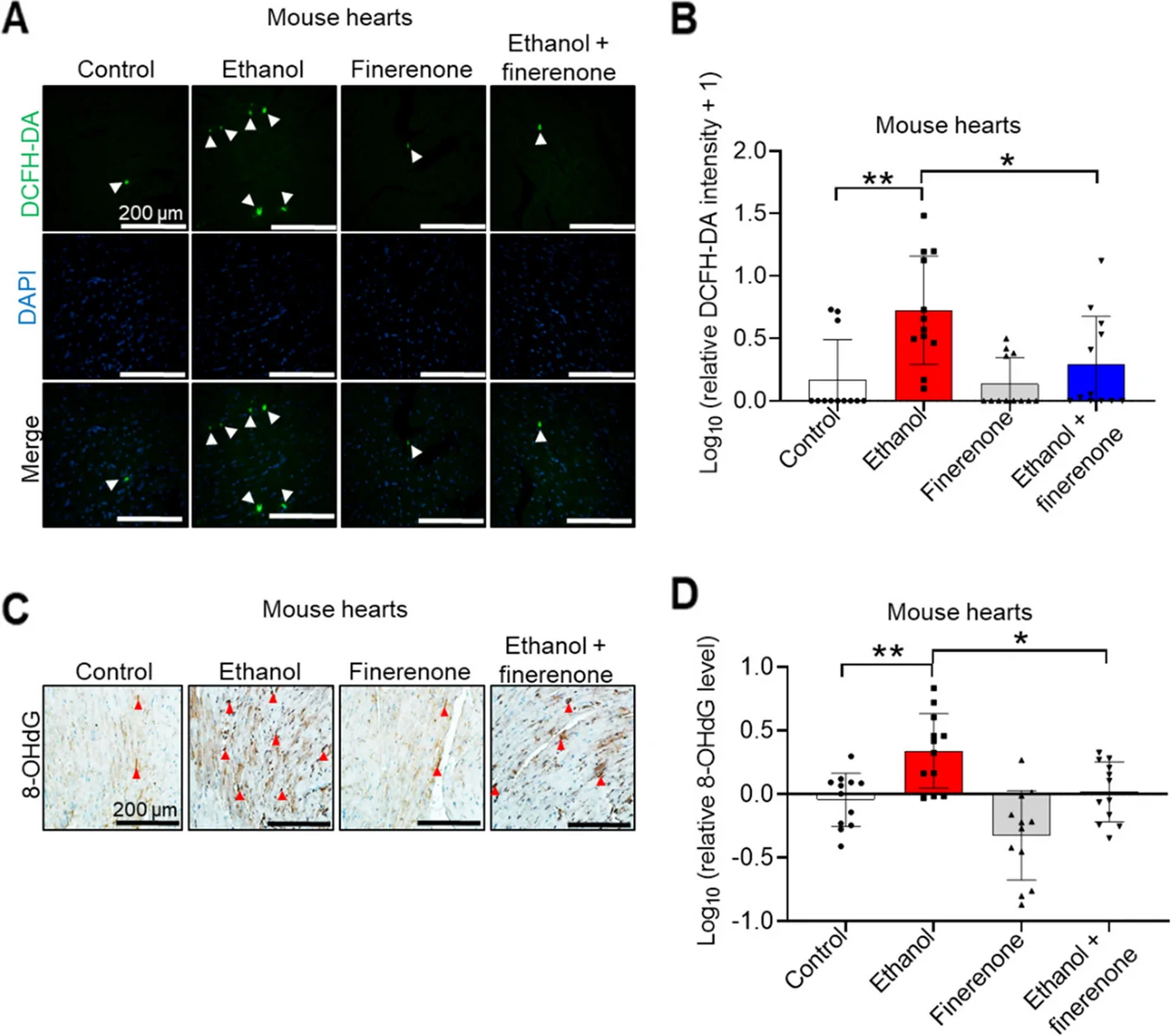
Finerenone attenuated the acute ethanol-induced cardiac oxidative stress in mice. **A** Myocardial tissue from mice treated with ethanol ± finerenone was analyzed using DCFH-DA immunofluorescence staining to assess oxidative stress levels. Scale bar = 200 μm. **B** Bar graph shows Log_10_ (relative DCFH-DA intensity + 1) per image field. *n* = 12 (four mice in each group, three images were analyzed for each mouse heart). Mean ± SD. *P < 0.05, **P < 0.01; two-way ANOVA. **C** Oxidative DNA damage was evaluated via immunohistochemical staining for 8-OHdG levels. Scale bar = 200 μm. **D** Bar graph shows the Log_10_ (relative 8-OHdG level) per image field. *n* = 12 (four mice in each group, three images were analyzed for each mouse heart). Mean ± SD. *P < 0.05, **P < 0.01; two-way ANOVA

MR-induced activation of NOXs has been well documented to promote oxidative stress (Wu et al., 2018). Specifically, NOX2-derived superoxide has been implicated in the development of ethanol cardiotoxicity (Brandt et al., 2016). To further validate the modulatory effects of finerenone on cardiac oxidative stress, mouse myocardial tissue was further analyzed using immunofluorescence for DCFH-DA staining. Results showed that finerenone significantly reduced ethanol-induced DCFH-DA signals in myocardial tissue (Fig. 6A and B). Additionally, oxidative DNA damage was assessed via 8-OHdG immunohistochemistry staining. Ethanol exposure significantly increased 8-OHdG intensity in myocardial tissue, while finerenone treatment markedly attenuated this ethanol-induced increase in 8-OHdG signals (Fig. 6C and D).

### Finerenone attenuated ethanol-induced mitochondrial dysregulation

**Fig. 7:**
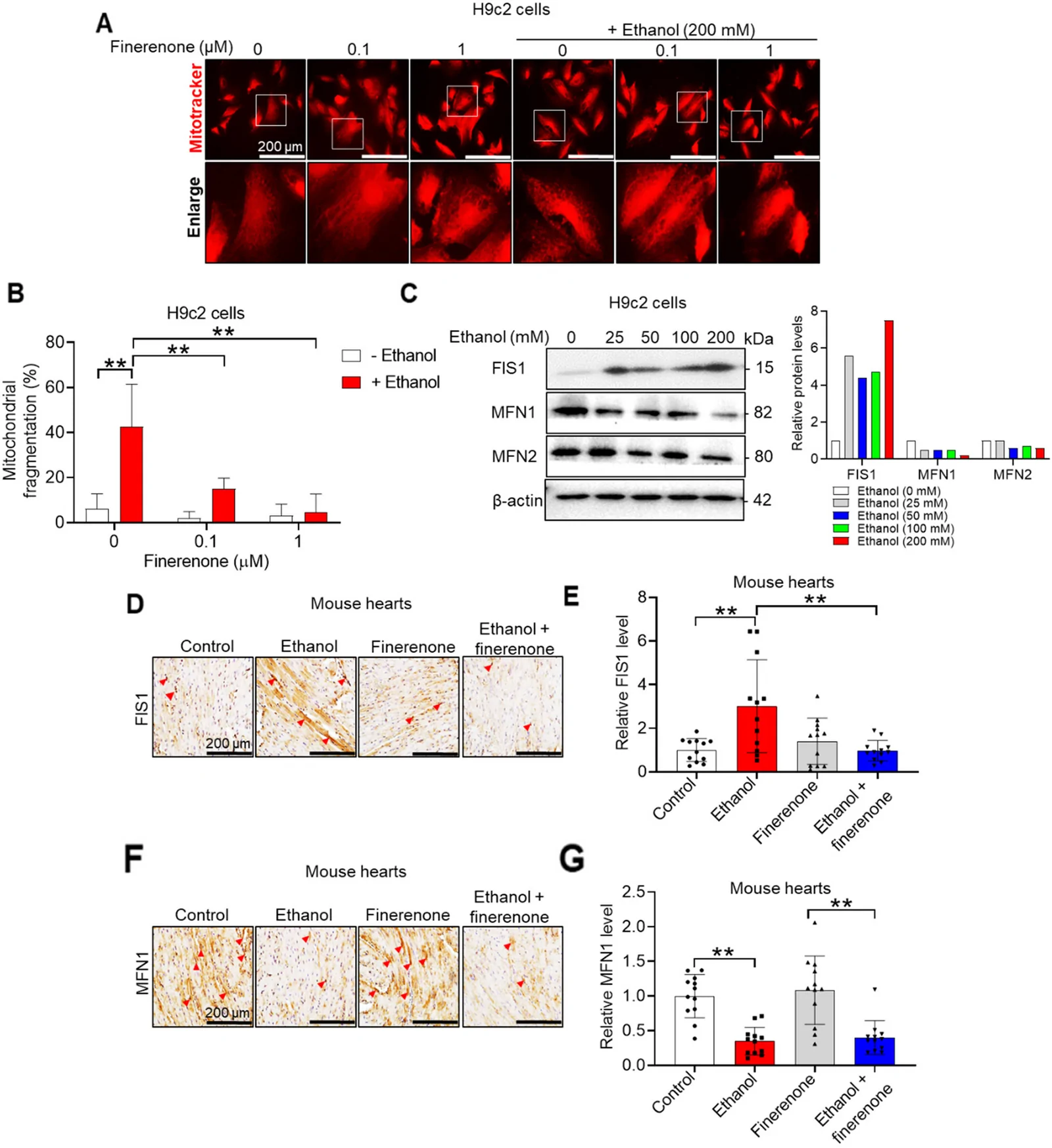
Finerenone attenuated ethanol-induced mitochondrial fission in H9c2 cardiomyocytes and mouse hearts. **A** H9c2 cells were treated with finerenone (0.1 and 1 μM) alone or in combination with ethanol (200 mM) for 24 h and stained with MitoTracker Red. Mitochondria were observed under a fluorescence microscope. The bottom panel shows an enlarged view of the cell indicated by the arrow in the top panel. Scale bar = 200 μm. **B** Bar graph shows the ratio of mitochondrial fragmentation (*n* = 5). Mean ± SD. **P < 0.01; two-way ANOVA. **C** Immunoblotting analysis of MFN1, MFN2, and FIS1 expression in H9c2 cells treated with ethanol (0–200 mM) for 24 h. Bar graph shows the relative levels of FIS1, MFN1, and MFN2. The experiments of Figs. 3A and 7C were performed using the same sample and blot, and the identical β-actin bands are shown in these two panels. Myocardial tissue from mice treated with ethanol ± finerenone was analyzed by immunohistochemistry to assess the expression of FIS1 (**D**) and MFN1 (**F**). Scale bar = 200 μm. Bar graphs show the relative intensity of (**E**) FIS1 and (**G**) MFN1, respectively. *n* = 12 (four mice in each group, three images were analyzed for each mouse heart). Mean ± SD. **P < 0.01; two-way ANOVA

Given that ethanol cardiotoxicity has been associated with mitochondrial dysfunction (Piano and Phillips 2014), targeting MR with finerenone may help mitigate ethanol-induced mitochondrial damage in cardiomyocytes. To assess the cardioprotective effects of finerenone against ethanol-induced mitochondrial damage, we utilized MitoTracker Red staining in H9c2 cells (Fig. 7A). Results showed that finerenone treatment alone did not affect mitochondrial fragmentation (Fig. 7A and B). Ethanol (200 mM) significantly increased mitochondrial fragmentation (Fig. 7A and B), whereas finerenone (0.1 and 1 μM) significantly reduced ethanol-induced mitochondrial fragmentation (Fig. 7B). Further analysis of mitochondrial fusion and fission-related genes revealed that ethanol dose-dependently suppressed the expression of fusion markers MFN1 and MFN2 while increasing the expression of the fission marker FIS1 in H9c2 cells (Fig. 7C). To further validate the modulatory effects of finerenone on cardiac mitochondria, mouse myocardial tissue was analyzed using immunohistochemistry staining for FIS1 and MFN1 expressions. The results showed that finerenone treatment significantly reduced ethanol-induced FIS1 signals in myocardial tissue (Fig. 7D and E). However, ethanol suppressed MFN1 expression, even when combined with finerenone treatment (Fig. 7F and G). These findings suggest that MR blockade with finerenone treatment mitigated ethanol-induced mitochondrial dysfunction primarily by involving mitochondrial fission rather than fusion.

## Discussion

Although several pathological mechanisms have been proposed, the contribution of ethanol-induced toxicity to multi-organ dysfunction and endocrine disturbances is well known (The effects of alcohol on physiological processes and biological development 2004). Ethanol and its metabolites exert direct cytotoxic effects on cardiomyocytes, triggering cellular damage and death (Wilke et al., 1996). This process releases damage-associated molecular patterns, such as mitochondrial DNA and high-mobility group box 1 (HMGB1), which activate neutrophils and M1 macrophages, initiating an inflammatory response to clear damaged cardiomyocytes and extracellular matrix debris and to transition toward tissue repair (Thomas and Grisanti 2020; Guo and Ren 2010b). However, during the inflammation-to-repair phase, pro-inflammatory cytokines such as TGF-β, IL-1β, and IL-6 promote fibroblast activation and differentiation into myofibroblasts, thereby contributing to extracellular matrix (ECM) remodeling and fibrosis (Thomas and Grisanti, 2020; Guo and Ren 2010b). Repeated cycles of inflammation and repair exacerbate progressive and ultimately irreversible fibrosis (Wynn and Ramalingam 2012). Additionally, after inflammation subsides, pro-inflammatory M1 macrophages are reprogrammed into anti-inflammatory M2 macrophages to support tissue repair and suppress fibrosis associated with fibroblast-to-myofibroblast transitions (Thomas and Grisanti 2020; Guo and Ren 2010b). An imbalance in the M1/M2 macrophage ratio plays a critical role in initiating and exacerbating lesions and fibrosis (Lis-Lopez et al., 2021). Panchenko et al. reported elevated circulating levels of cytokines, including IL-6, IL-8, IL-12, TNF-α, and its soluble receptor, s-TNF-R, along with a twofold increase in C-reactive protein levels in patients with ACM (Panchenko et al., 2015). Similarly, Mouton et al. demonstrated that chronic alcohol exposure activates pro-inflammatory cardiac fibroblasts, leading to the release of cytokines such as TNF-α, IL-6, IL-1β, and IL-33 (Mouton et al., 2020). Zagrosek et al. observed that binge drinking induces transient myocarditis-like inflammation detectable via cardiac magnetic resonance imaging (Zagrosek et al., 2010). Inflammation is well-established as a key driver of cardiac fibrosis (Thomas and Grisanti 2020; Kania et al., 2009). Given that ACM becomes irreversible once cardiac fibrosis develops (Andersson et al., 2022; Prasad and Halliday 2021), resolving alcohol-induced inflammation is essential to prevent ACM progression and irreversible fibrosis. Thus, developing therapeutic strategies that attenuate cardiomyocyte injury and dampen inflammation to limit fibrosis is critical in improving ACM outcomes. We also found that an acute ethanol challenge significantly upregulated soluble IL-1β, HMGB1, and soluble TNF-α in mouse hearts, and that oral finerenone significantly blocked the ethanol-induced local cytokine storms. The data supported that finerenone may act as a potent anti-inflammatory agent against acute alcoholic myocardial injury.

In this study, we found that finerenone attenuates ethanol-induced oxidative stress, apoptosis, and inflammation by suppressing the expression of IL-1β, HMGB1, and iNOS. Given that MR is broadly expressed in the cardiovascular system and is known to regulate oxidative stress, inflammation, vasoconstriction, vascular remodeling, and fibrosis (Belden et al., 2017), our findings demonstrate that finerenone-mediated MR antagonism confers antioxidative, anti-apoptotic, and anti-inflammatory effects. These results are consistent with previous studies, which have shown that cardiac MR deletion reduces oxidative stress, apoptosis, and fibrosis, whereas MR overexpression induces dilated cardiomyopathy (Bauersachs and Lopez-Andres 2022). Antagonizing MR with finerenone ameliorated ethanol-induced oxidative stress, inflammation, and apoptosis, supporting the hypothesis that targeting MR is an effective strategy against ethanol cardiotoxicity. The anti-inflammatory effect of finerenone was also confirmed by its ability to suppress ethanol-induced activation of the pro-inflammatory NF-κB p65 in cardiomyocytes. Furthermore, MR-induced activation of NOXs is implicated in macrophage polarization (Yunna et al., 2020). Given that macrophages are well known for regulating fibroblast activity involved in ECM remodeling and fibrosis, promoting the differentiation and activation of cardiac fibroblasts is recognized as a pivotal process driving ECM remodeling and cardiac fibrosis (Thomas and Grisanti 2020; Guo and Ren 2010b). Accordingly, blocking MR may prevent downstream fibrotic remodeling. Jin et al. reported that finerenone mitigated myocardial fibrosis in a rat model of type 2 diabetes (Jin et al., 2023). Similarly, Lavall et al. demonstrated that finerenone mitigated aldosterone-induced cardiac fibrosis by blocking MR-induced upregulation of CTGF, TGF-β, and lysyl oxidase in cardiac fibroblasts (Lavall et al., 2019). Our results showed that finerenone mitigated ethanol-induced macrophage infiltration in the cardiac tissue. In line with our findings, Fraccarollo et al. reported that a specific MR deficiency in macrophages prevents macrophage infiltration, inflammation, fibrosis, and oxidative stress in the hearts of aged mice (Fraccarollo et al., 2024). Additionally, Barrera-Chimal et al. demonstrated that finerenone promotes activation of IL-4 signaling and polarizes macrophages toward the M2 phenotype (Barrera-Chimal et al., 2018). These findings collectively support the idea that anti-inflammatory and anti-fibrotic activities may contribute to the cardioprotective mechanism of finerenone against ethanol cardiotoxicity.

Moreover, mitochondrial dysfunction, which leads to oxidative stress, cell death, and unresolved inflammation, has been recognized as a significant contributor to ACM pathogenesis (Wilke et al., 1996; Obad et al., 2018; Leclercq et al., 2017). Ethanol-induced mitochondrial dysfunction is primarily implicated in disrupting fusion/fission dynamics, leading to mitochondrial fragmentation and stress (Eisner et al., 2017; Oh et al., 2020). Eisner et al. reported that ethanol exposure inhibits mitochondrial fusion (Obad et al., 2018), while Oh et al. reported that ethanol induces mitochondrial fragmentation primarily mediated by increased mitochondrial fission (Oh et al., 2020). Our findings confirm that ethanol induces mitochondrial fragmentation through both increased fission and decreased fusion, as indicated by inverse expression patterns of FIS1 (fission marker) and MFNs (fusion markers) in ethanol-treated H9c2 cardiomyocytes. Notably, finerenone attenuated ethanol-induced FIS1 upregulation in cardiac tissue, though it had no significant effect on MFN1 levels. These results suggest that ethanol disrupts mitochondrial dynamics in part through MR signaling, primarily by enhancing mitochondrial fission. Our results showed that finerenone prevents cardiac mitochondrial fragmentation and oxidative stress during ethanol exposure. Finerenone mitigated ethanol-induced oxidative stress, reduced DCFH-DA staining in ethanol-treated H9c2 cardiomyocytes, and decreased the accumulation of the DNA oxidation product 8-OHdG in ethanol-treated mouse hearts. These findings suggest that MR antagonism with finerenone can prevent mitochondrial fragmentation and oxidative stress, thereby attenuating cytokine storms in the acute ethanol cardiotoxicity model.

Circulating aldosterone has been reported to increase in response to ethanol stimulation and contribute to cardiovascular remodeling (Sobrino et al., 2019; Aoun et al., 2018). Consistent with this, our results showed that the ethanol-induced increase in aldosterone was detectable in myocardial tissue. However, treatment with finerenone only mildly attenuated ethanol-induced cardiac aldosterone levels, and this reduction did not reach statistical significance. Notably, MR activation can occur in a ligand-independent manner via Rac1, which itself is activated by oxidative stress. Once activated, MR further promotes the generation of reactive oxygen species (ROS) (Pan et al., 2024; Nagase and Fujita 2013; Barrera-Chimal and Jaisser 2019). This establishes a feed-forward loop between Rac1 and oxidative stress, amplifying MR activation and ROS production, thereby exacerbating cardiac injury (Nagase et al., 2012). Reportedly, rat myocardial tissues can produce endogenous aldosterone (Takeda et al., 2000; White 2003; Silvestre et al., 1999). H9c2 cells used in this study are a rat myocardial cell line, and the aldosterone-dependent or– independent pathways should be further studied in myocardial cells and tissues after treatment with ethanol ± finerenone in the future. Grune et al. reported that finerenone blocked isoproterenol-induced cardiac fibrosis and macrophage infiltration despite elevated compensatory serum aldosterone levels (Grune et al., 2018). Moreover, they noted that finerenone exhibited anti-fibrotic and anti-inflammatory effects, which were not observed in the spironolactone-treated group (Grune et al., 2018). This preclinical study supported the view that finerenone may have a more potent cardioprotective effect than spironolactone. Additionally, finerenone exhibits greater MR selectivity and a lower incidence of hyperkalemia-related treatment discontinuation compared with traditional steroidal MRAs in certain clinical settings (Di Lullo et al., 2023). However, direct evidence demonstrating superiority over spironolactone or eplerenone in the general heart failure population remains limited. Therefore, the potential of finerenone and steroid MRAs to combat ethanol cardiotoxicity should be further compared through side-by-side comparisons.

In this study of acute myocardial injury, a 3-day ethanol exposure (3 g/kg/d) significantly promoted myocardial apoptosis, and a previous study also reported that this acute treatment can induce Bcl-2/Bax-dependent caspase-3 activation by modulating PDCD4 and PI3K/Akt pathways (Deng et al., 2021). Interestingly, ethanol-induced autophagy also promotes myocardial injury in this acute alcoholic myocardial injury model (Guo and Ren 2012a; Tao et al., 2021). Thus, the effect of finerenone on myocardial autophagy should be further investigated using this acute myocardial injury model in the future. In cell experiments, ethanol treatment at 200 mM for 24 h significantly reduced cell viability in H9c2 cells, consistent with a previous study (Shi et al., 2016). Additionally, many studies use 200 mM ethanol concentration as a standard for inducing cell damage in H9c2 cells (Tao et al., 2021; Deng et al., 2021; Tian et al., 2021, 2023; Meng et al., 2024). Moreover, the ethanol metabolite acetaldehyde plays a major role in the development of alcoholic myocardial injury, and alcohol dehydrogenase (ADH) and aldehyde dehydrogenase-2 (ALDH2), as key enzymes, participate in ethanol metabolism (Brandt et al., 2016; Li et al., 2009; Guo et al., 2012b). The cardioprotective effect of finerenone should be further validated in acetaldehyde-treated cardiomyocytes in the future. For *in vitro* assays, the concentrations of finerenone used in the present study were selected based on clinically relevant pharmacokinetic data (Heinig and Eissing 2023; Heinig et al., 2018). Previous phase I pharmacokinetic studies demonstrated that oral administration of finerenone in healthy volunteers resulted in peak plasma concentrations (Cmax) of approximately 80–160 ng/mL at clinically used doses (Heinig and Eissing 2023). Given the molecular weight of finerenone (378.45 g/mol), these concentrations correspond to approximately 0.2–0.4 μM. Furthermore, dose-proportional pharmacokinetics have been reported across a dose range of 1.25–80 mg, with plasma concentrations approaching the micromolar range at higher exposures (Heinig et al., 2018). Therefore, the concentrations of 0.1 to 1 μM used in the present *in vitro* experiments were considered pharmacologically and clinically relevant, encompassing the range of systemic exposure achievable in humans while allowing evaluation of dose-dependent biological effects. Furthermore, the peak plasma concentration achieved with 10 mg/kg finerenone in rodents is approximately 75 ng/mL (~ 0.2 μM) (Nachimuthu et al., 2025), which falls within the concentration range (0.1 to 1 μM) used in the present *in vitro* experiments. 10 mg/kg finerenone showed a potent cardioprotective effect in this acute alcoholic myocardial injury mouse model, and previous studies using various cardiovascular disease mouse models also supported that 10 mg/kg is a suitable dose for finerenone-related animal research (Grune et al., 2016, 2018; Morikawa et al., 2026).

In this study, we found that acute ethanol exposure induced MR upregulation, thereby further promoting iNOS overexpression, cytokine storms, oxidative stress, and mitochondrial dysregulation in myocardial cells and tissues. Blocking MR signaling with finerenone significantly reduced ethanol-induced myocardial injury by attenuating iNOS expression, the inflammatory response, oxidative DNA damage, and mitochondrial fragmentation. Collectively, we demonstrated for the first time that finerenone may serve as a potent and novel cardioprotective agent against acute alcoholic myocardial injury. The major limitation of this study is the lack of echocardiographic or hemodynamic measurements in mice, which are crucial for establishing the translational validity of this study from histological changes to functional assessment. Serum CK-MB levels and histological endpoints (such as TUNEL assay for apoptosis and iNOS/F4/80 for inflammation) serve as highly sensitive, reliable, and well-established clinical and preclinical indicators to directly reflect acute cardiomyocyte damage and myocardial necrosis (Jaffe et al., 2006; Tan et al., 2012), but assessment of cardiac function mandates serial, dynamic, and non-invasive echocardiographic measurements, not just histological or molecular changes. In the present study, a 3-day administration with ethanol ± finerenone significantly caused molecular and histological changes, including serum CK-MB level (a myocardial injury marker), cardiac cell death, oxidative stress, and myocardial inflammation in mice, but the effect of these molecular/histological changes on actual cardiac functions was still unclear. This study should be applied in clinical settings with caution because the effects of finerenone on the pathological characteristics of ACM, including cardiomegaly, cardiac fibrosis, and chamber dilatation, have not been addressed. Since our study used an acute animal model with binge alcohol administration, the therapeutic potential of finerenone in ACM, which is primarily a chronic disease, should be evaluated using chronic data. A previous study also found that 3 g/kg of ethanol by intraperitoneal injection can induce acute alcoholic liver injury in mice (Wang et al., 2018). Therefore, the effects of finerenone on ethanol hepatotoxicity should also be studied.

## Conclusion

This study demonstrates that finerenone attenuates acute alcohol-induced myocardial injury by suppressing inflammation, oxidative stress, macrophage infiltration, and mitochondrial fragmentation. These findings suggest that finerenone may serve as a promising cardioprotective agent for alcohol-related myocardial damage. Further studies using chronic alcohol exposure models and functional cardiac assessments are needed to confirm its translational and clinical relevance.

## Supplementary Information

Supplementary Material 1.

Supplementary Material 2.

## Data Availability

Data will be made available on request.

## Abbreviations

ACM: Alcoholic cardiomyopathy
MRA: Mineralocorticoid receptor antagonist
IL: Interleukin
TNFα: Tumor necrosis factor α
TGF-β: Transforming growth factor β
