# Supplementary material for: Finerenone mitigates acute alcoholic myocardial injury by modulating inflammatory signaling, oxidative stress, and mitochondrial function
Source: Mol Med. 2026 Jun 20;32:141. doi: 10.1186/s10020-026-01532-2 (PMC13560241; doi:10.1186/s10020-026-01532-2)

Finerenone Mitigates Acute Alcoholic Myocardial Injury by Modulating Inflammatory Signaling, Oxidative Stress, and Mitochondrial Function

Original Image for Western Blots

Fig. 1E

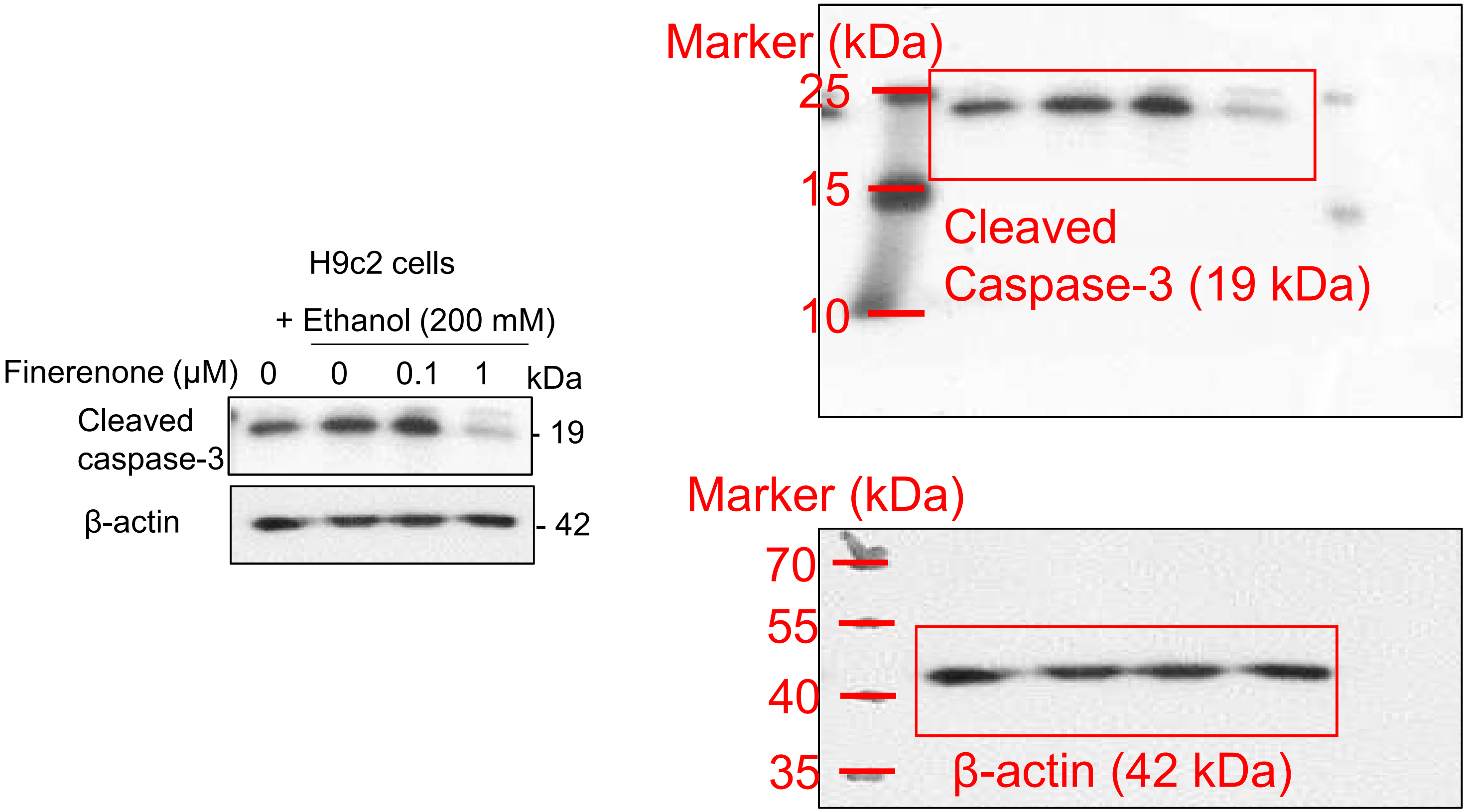

H9c2 cells
+ Ethanol (200 mM)
Finerenone (μM) 0 0 0.1 1 kDa
Cleaved caspase-3
19
β-actin
42
Marker (kDa)
25
15
10
Cleaved Caspase-3 (19 kDa)
Marker (kDa)
70
55
40
35
β-actin (42 kDa)

Fig. 3A

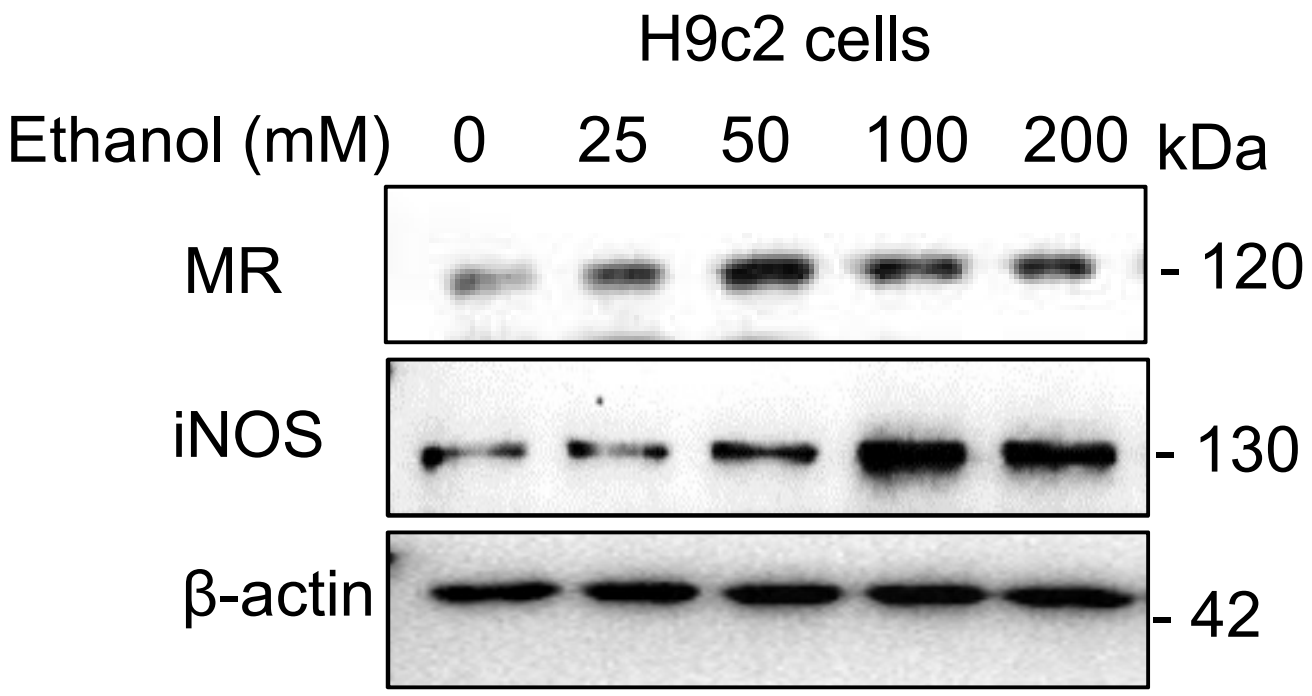

H9c2 cells
Ethanol (mM) 0 25 50 100 200 kDa
MR
- 120
iNOS
- 130
β-actin
- 42

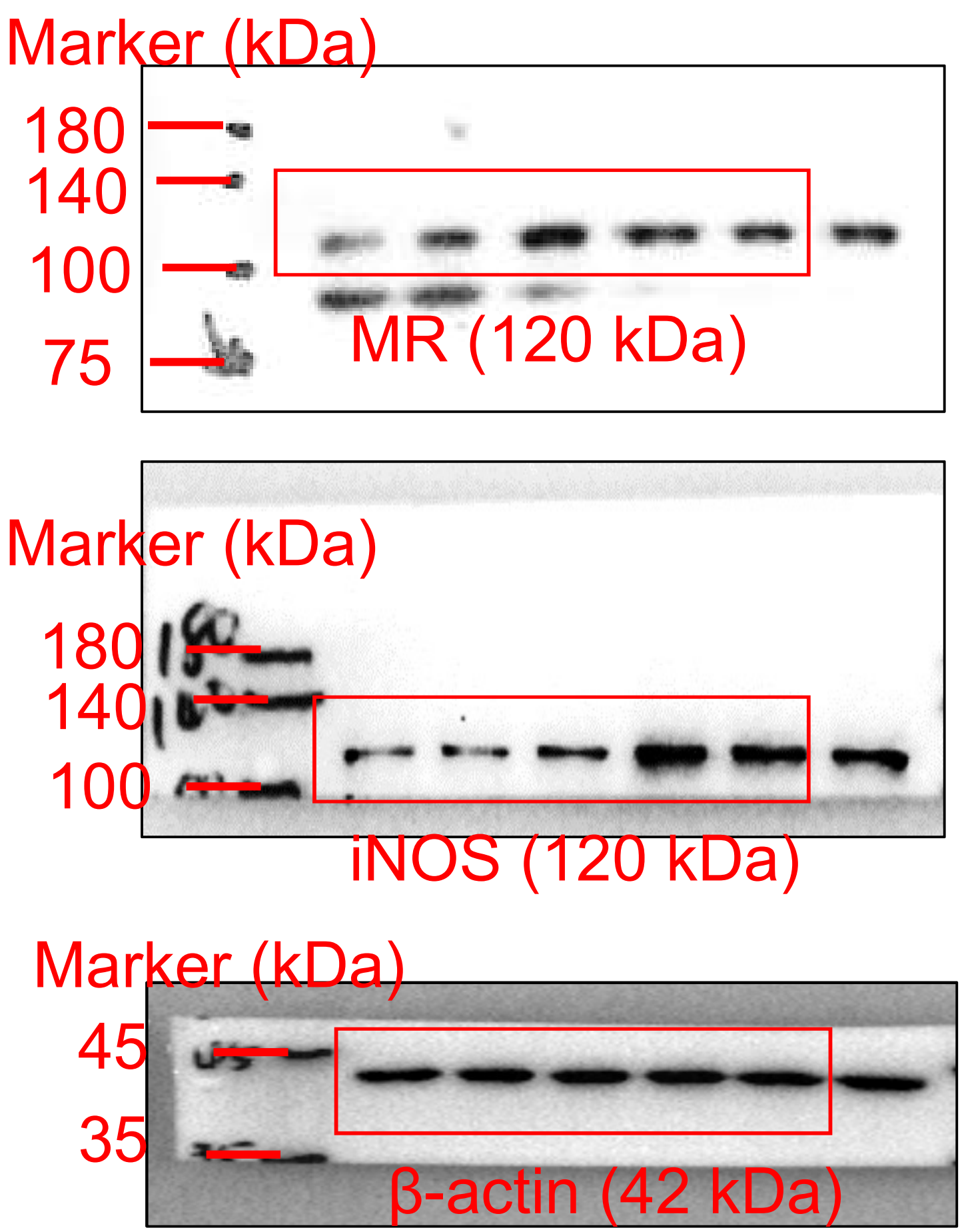

Marker (kDa)
180
140
100
75
MR (120 kDa)
Marker (kDa)
180
140
100
iNOS (120 kDa)
Marker (kDa)
45
35
β-actin (42 kDa)

Fig. 3B

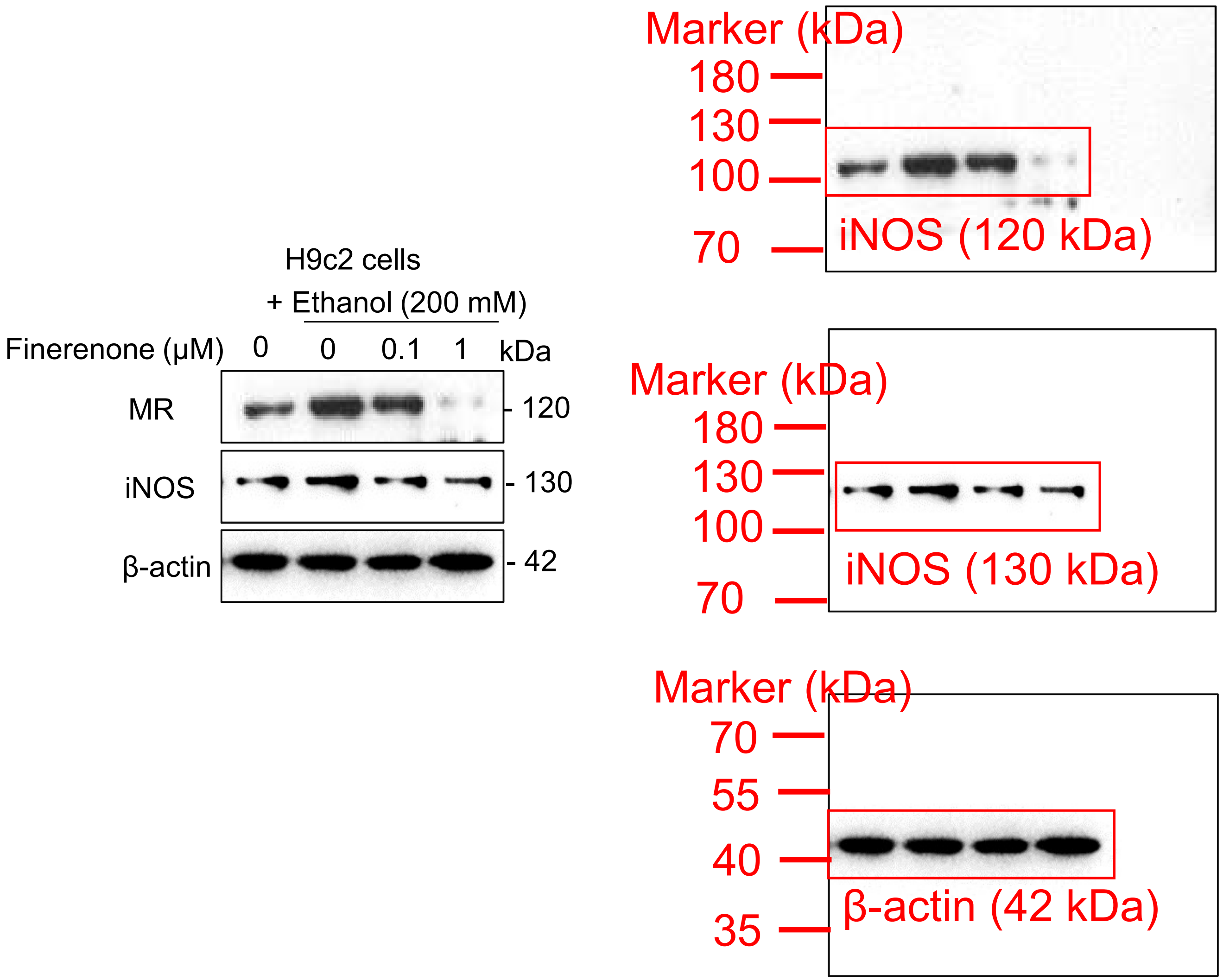

H9c2 cells
+ Ethanol (200 mM)
Finerenone (μM) 0 0 0.1 1 kDa
MR
- 120
iNOS
- 130
β-actin
- 42
Marker (kDa)
180
130
100
70
iNOS (120 kDa)
Marker (kDa)
180
130
100
70
iNOS (130 kDa)
Marker (kDa)
70
55
40
35
β-actin (42 kDa)

Fig. 3E

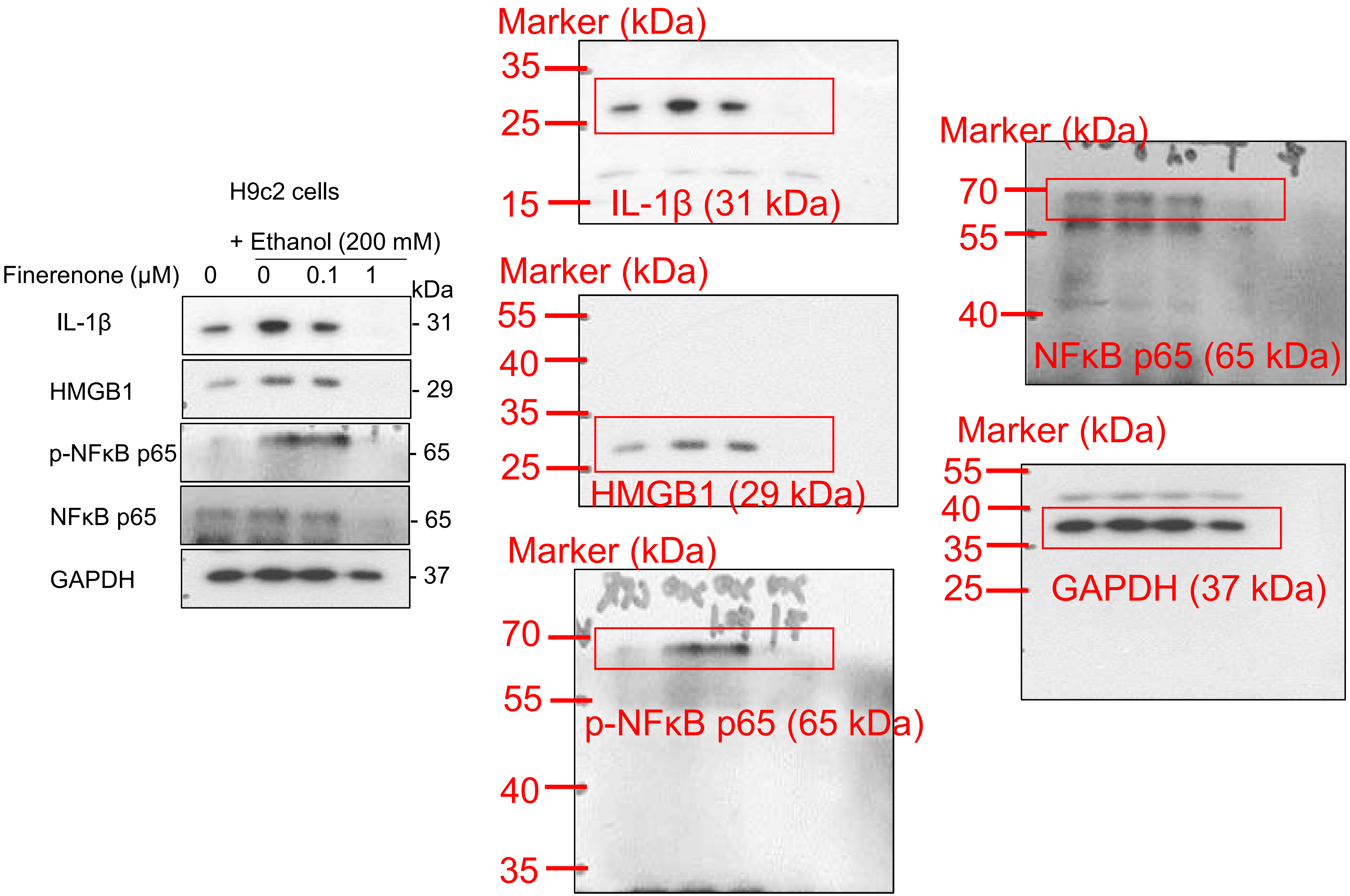

H9c2 cells
+ Ethanol (200 mM)
Finerenone (μM) 0 0 0.1 1
kDa
IL-1β - 31
HMGB1 - 29
p-NFκB p65 - 65
NFκB p65 - 65
GAPDH - 37
Marker (kDa)
35
25
15
IL-1β (31 kDa)
Marker (kDa)
55
40
35
25
HMGB1 (29 kDa)
Marker (kDa)
70
55
40
35
p-NFκB p65 (65 kDa)
Marker (kDa)
70
55
40
NFκB p65 (65 kDa)
Marker (kDa)
55
40
35
25
GAPDH (37 kDa)

Fig. 4G

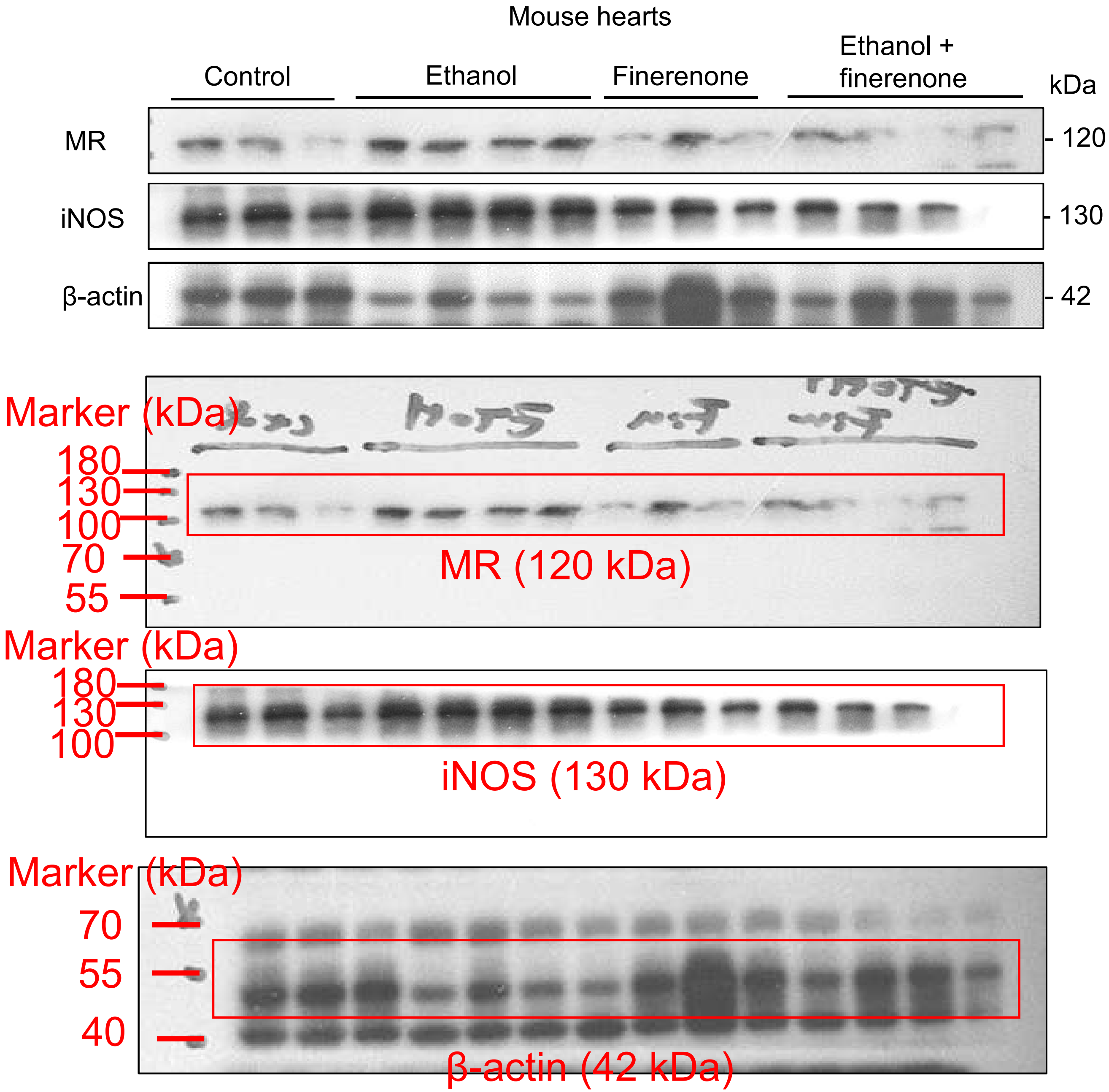

Mouse hearts
Control
Ethanol
Finerenone
Ethanol + finerenone
kDa
MR
iNOS
β-actin
- 120
- 130
- 42
Marker (kDa)
180
130
100
70
55
MR (120 kDa)
Marker (kDa)
180
130
100
iNOS (130 kDa)
Marker (kDa)
70
55
40
β-actin (42 kDa)

Fig. 5A

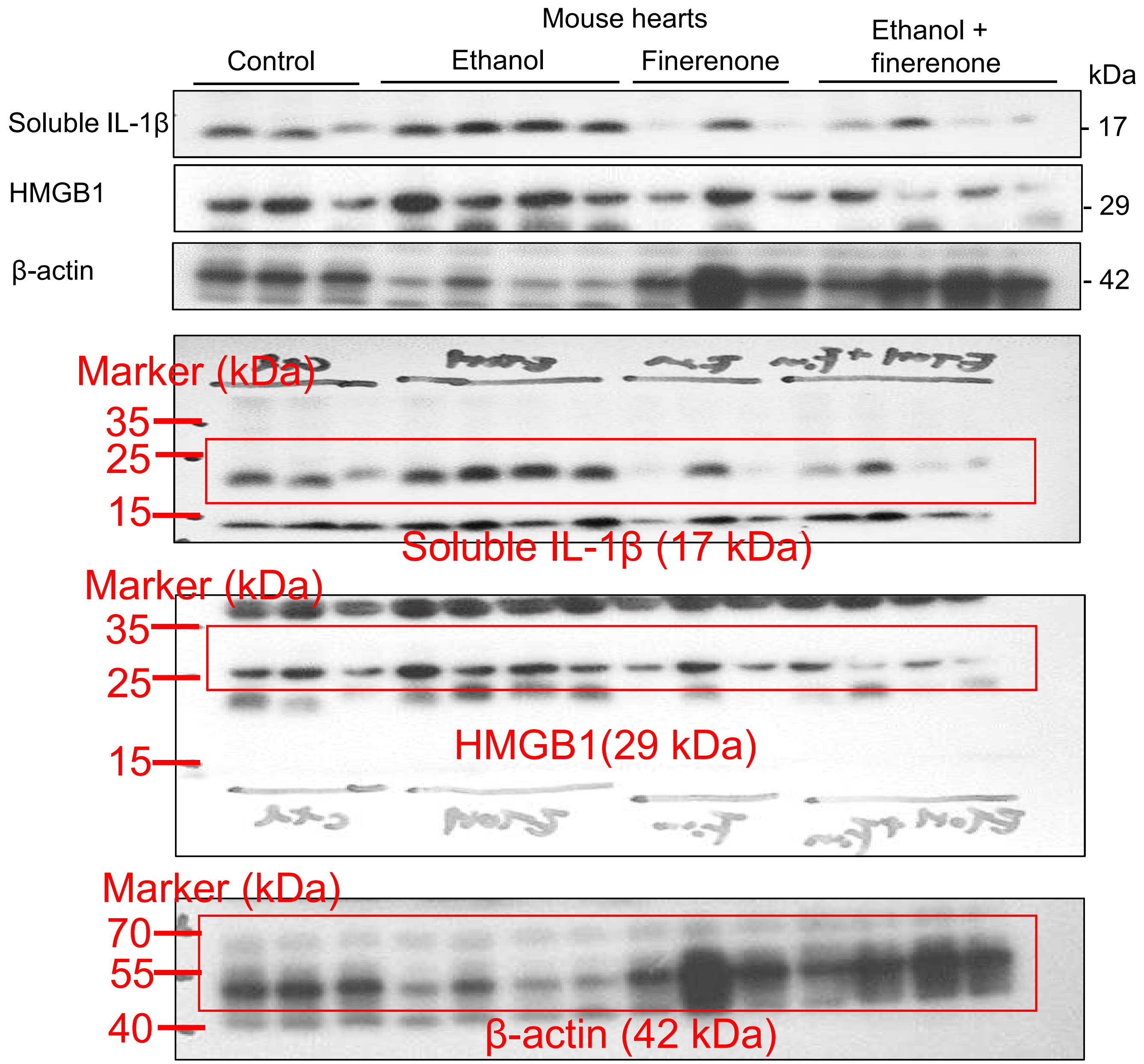
Mouse hearts
Control
Ethanol
Finerenone
Ethanol + finerenone
kDa
Soluble IL-1β
HMGB1
β-actin
17
29
42
Marker (kDa)
35
25
15
Soluble IL-1β (17 kDa)
Marker (kDa)
35
25
15
HMGB1(29 kDa)
Marker (kDa)
70
55
40
β-actin (42 kDa)

Fig. 7C

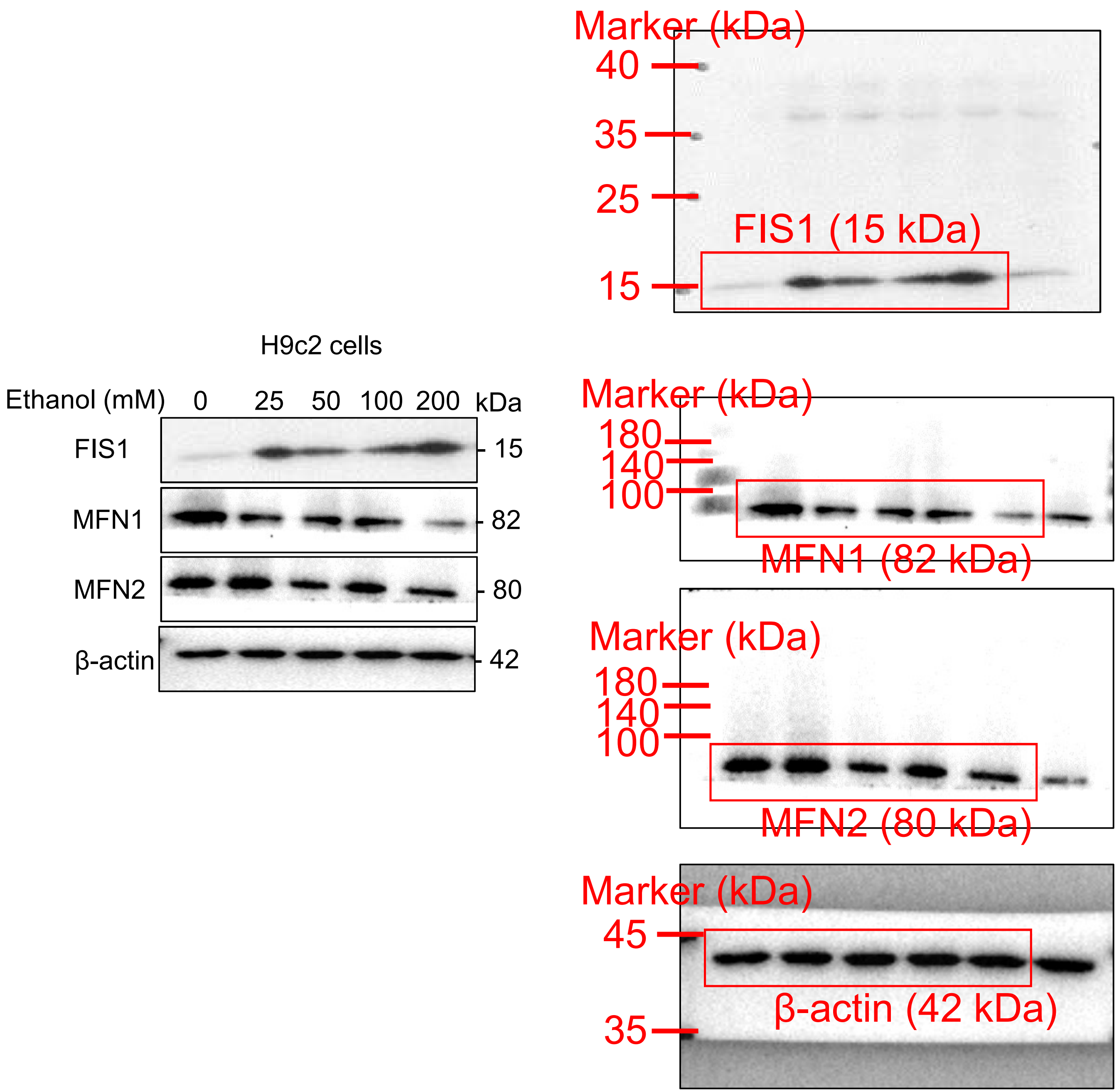

H9c2 cells
Ethanol (mM) 0 25 50 100 200 kDa
FIS1 15
MFN1 82
MFN2 80
β-actin 42
Marker (kDa)
40
35
25
15
FIS1 (15 kDa)
Marker (kDa)
180
140
100
MFN1 (82 kDa)
Marker (kDa)
180
140
100
MFN2 (80 kDa)
Marker (kDa)
45
35
β-actin (42 kDa)

Supplementary Fig. 1

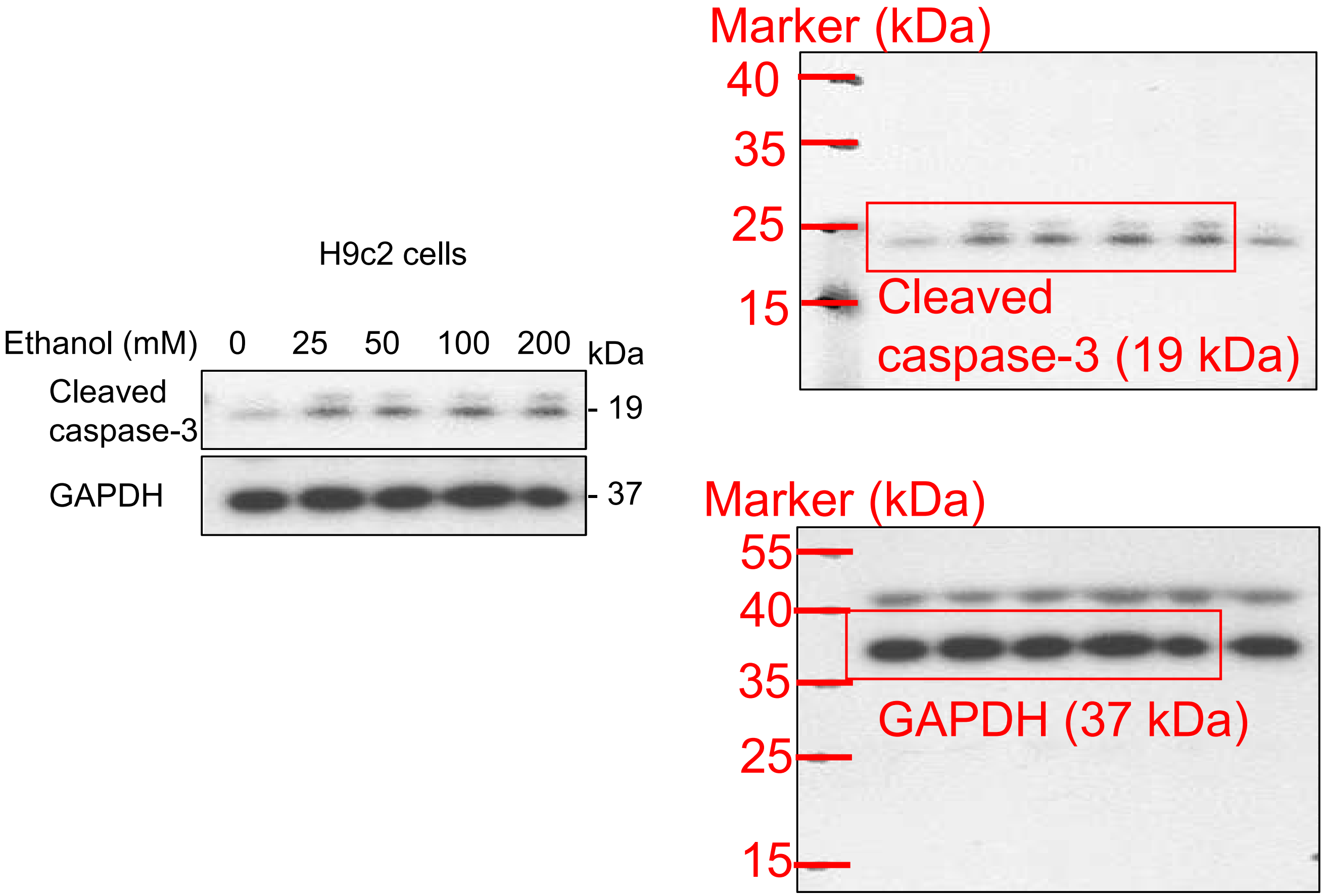

Supplementary Fig. 2

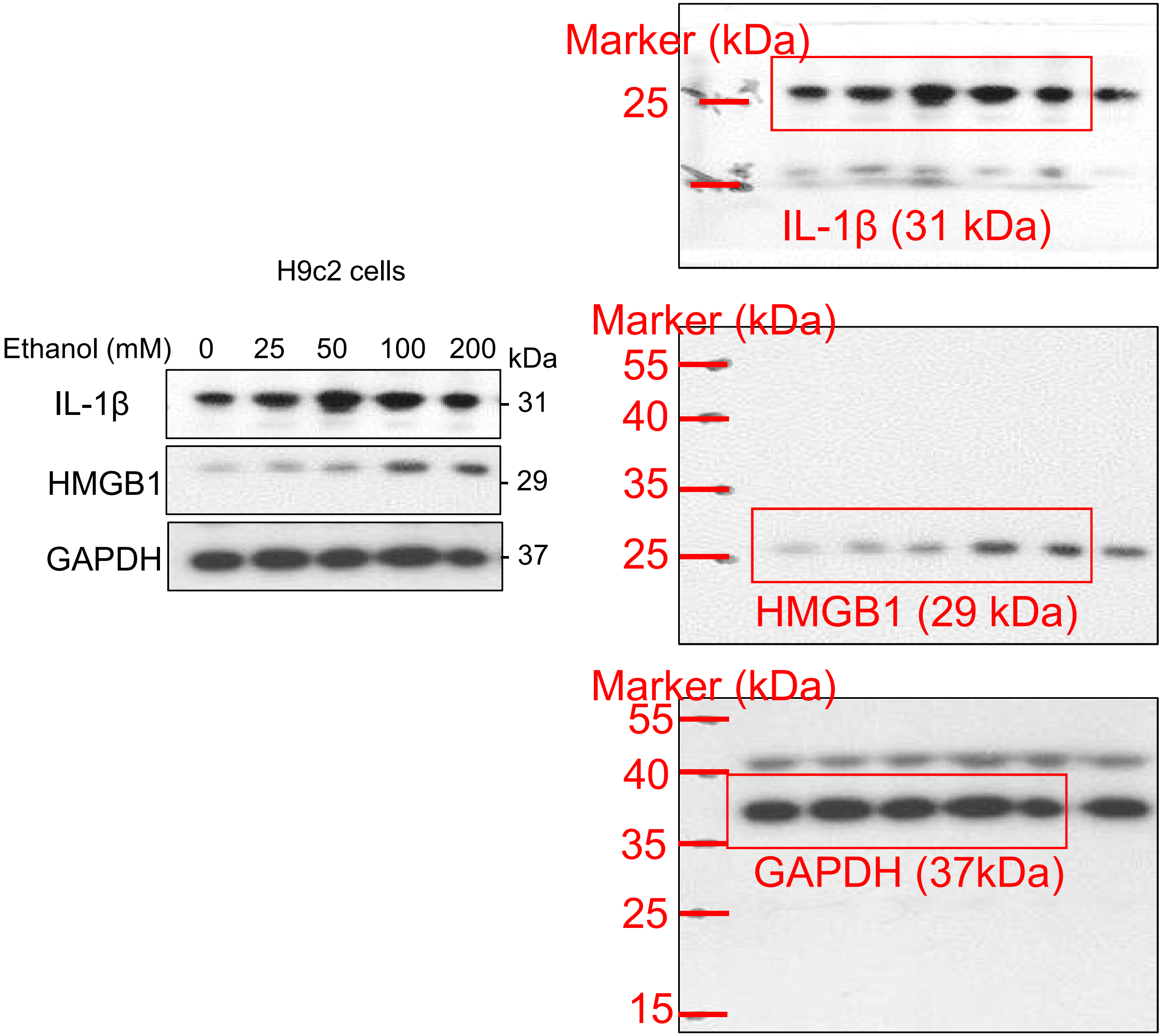

Supplementary Fig. S3

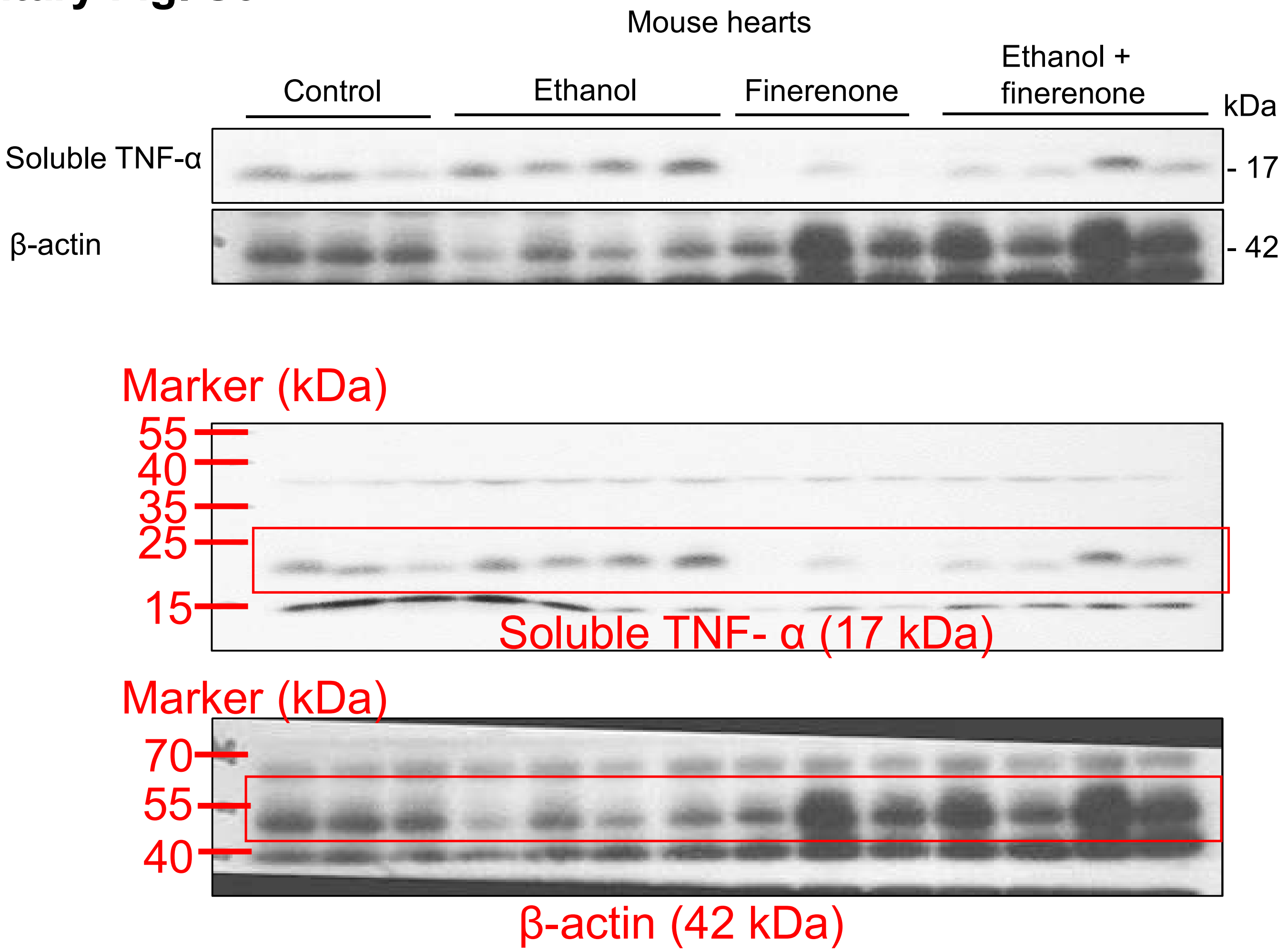

Supplement: Supplementary file 1 [file 10020_2026_1532_MOESM2_ESM.pdf]
