# Supplementary material for: Finerenone mitigates acute alcoholic myocardial injury by modulating inflammatory signaling, oxidative stress, and mitochondrial function
Source: Mol Med. 2026 Jun 20;32:141. doi: 10.1186/s10020-026-01532-2 (PMC13560241; doi:10.1186/s10020-026-01532-2)
Supplement: Supplementary file 2 [file 10020_2026_1532_MOESM1_ESM.docx]

**Finerenone Mitigates Acute Alcoholic Myocardial Injury by Modulating Inflammatory Signaling, Oxidative Stress, and Mitochondrial Function**

**Authors**

Ching-Han Liu^1,2,3^, Tian-Huei Chu^4,5,6^, Shih-Ming Yang^7^, Yi-Chen Wang^1,6^, Wen-Chin Lee^8^, Yi-Chen Chang^4^, Yao-Chang Chen^9^, Yi-Jen Chen^10,11,12^, Cheng-Chieh Fang^4^, Zhi-Hong Wen^3,13,14^, Chien-Wei Feng^4,15^, Hao-Ming Wu^1^, Po-Tseng Lee^16^, Nan-Chieh Huang^17,18^, Shih-Chung Huang^1,2,6,19,20*^

**Author affiliations**

^1^Department of Internal Medicine, Division of Cardiology, Kaohsiung Armed Forces General Hospital, Kaohsiung, Taiwan.

^2^Department of Internal Medicine, Division of Cardiology, Tri-Service General Hospital, National Defense Medical University, Taipei, Taiwan

^3^Department of Marine Biotechnology and Resources, National Sun Yat-Sen University, Kaohsiung, Taiwan.

^4^Medical Laboratory, Medical Education and Research Center, Kaohsiung Armed Forces General Hospital, Kaohsiung, Taiwan.

^5^School of Medicine, National Defense Medical University, Taipei, Taiwan.

^6^Institute of Medical Science and Technology, National Sun Yat-sen University, Kaohsiung, Taiwan.

^7^Division of Plastic Surgery, Department of Surgery, Kaohsiung Medical University Hospital, Kaohsiung, Taiwan.

^8^Department of Internal Medicine, Division of Nephrology, Kaohsiung Chang Gung Memorial Hospital and Chang Gung University College of Medicine, Kaohsiung, Taiwan.

^9^Department of Biomedical Engineering, National Defense Medical University, Taipei, Taiwan.

^10^Graduate Institute of Clinical Medicine, College of Medicine, Taipei Medical University, Taipei, Taiwan.

^11^Division of Cardiology, Department of Internal Medicine, School of Medicine, College of Medicine, Taipei Medical University, Taipei, Taiwan.

^12^Division of Cardiovascular Medicine, Department of Internal Medicine, Wan Fang Hospital, Taipei Medical University, Taipei, Taiwan.

^13^Institute of BioPharmaceutical Sciences, National Sun Yat-sen University, Kaohsiung, Taiwan.

^14^National Museum of Marine Biology and Aquarium, Pingtung County, Taiwan.

^15^Center for Cancer Research, Kaohsiung Medical University, Kaohsiung, Taiwan.

^16^Division of Cardiology, Department of Internal Medicine, National Cheng Kung University Hospital, College of Medicine, National Cheng Kung University, Tainan, Taiwan.

^17^Diving Medical and Physiology Training Center, Zuoying Armed Forces General Hospital, Kaohsiung, Taiwan.

^18^Department of Information Engineering, I-Shou University, Kaohsiung, Taiwan.

^19^Department of Internal Medicine, Division of Cardiology, Pingtung Branch of Kaohsiung Armed Forces General Hospital, Pingtung, Taiwan.

^20^Department of Nursing, Fooyin University, Kaohsiung, Taiwan.

***Correspondence**

Shih-Chung Huang, M.D., Ph.D.

Department of Internal Medicine, Division of Cardiology, Pingtung Branch of Kaohsiung Armed Forces General Hospital, No. 22, Ln. 58, Dahu Rd., Pingtung City, Pingtung County 90092, Taiwan.

Telephone: +886-8-756-0756, extension: 774815

E-mail: sghung@gmail.com

**Supplementary Fig. 1. Ethanol treatment promoted the upregulation of cleaved caspase-3 in the cardiomyocytes.** The immunoblot analysis for cleaved caspase-3 in H9c2 cells after ethanol (25 to 200 μM) treatment for 24 h. Bar graph shows the relative cleaved-caspase-3 level.

**Supplementary Fig. 2. Proinflammatory genes were upregulated in the cardiomyocytes after ethanol stimulation.** The immunoblot analysis for IL-1β and HMGB1 in H9c2 cells after ethanol (25 to 200 μM) exposure for 24 h. Bar graph shows the relative levels of IL-1β and HMGB1.

**Supplementary Fig. 3. Oral finerenone blocked the acute ethanol-promoted TNF-α signaling in mouse cardiac tissues.** (A) The immunoblotting analysis for TNF-α expression was performed in mouse hearts after ethanol ± finerenone administration.　 3-4 mouse hearts per group were used for immunoblotting. (B) Bar graph shows the relative soluble TNF-α levels (n = 3 in the control group, n = 4 in the ethanol group, n = 3 in the finerenone group, and n = 4 in the ethanol + finerenone group). Mean ± SD. ***P* < 0.01; two-way ANOVA.
